# Paralogs of the Calcium-Dependent Activator Protein for Secretion Differentially Regulate Synaptic Transmission and Peptide Secretion in Sensory Neurons

**DOI:** 10.3389/fncel.2018.00304

**Published:** 2018-09-11

**Authors:** Ali H. Shaib, Angelina Staudt, Ali Harb, Margarete Klose, Ahmed Shaaban, Claudia Schirra, Ralf Mohrmann, Jens Rettig, Ute Becherer

**Affiliations:** ^1^Department of Cellular Neurophysiology, Center for Integrative Physiology and Molecular Medicine (CIPMM), Saarland University, Homburg, Germany; ^2^ZHMB Junior Group, Center for Integrative Physiology and Molecular Medicine (CIPMM), Saarland University, Homburg, Germany

**Keywords:** DRG neurons, CADPS, exocytosis, priming, large dense core vesicle, synaptic transmission, neuropeptide

## Abstract

The two paralogs of the calcium-dependent activator protein for secretion (CAPS) are priming factors for synaptic vesicles (SVs) and neuropeptide containing large dense-core vesicles (LDCVs). Yet, it is unclear whether CAPS1 and CAPS2 regulate exocytosis of these two vesicle types differentially in dorsal root ganglion (DRG) neurons, wherein synaptic transmission and neuropeptide release are of equal importance. These sensory neurons transfer information from the periphery to the spinal cord (SC), releasing glutamate as the primary neurotransmitter, with co-transmission via neuropeptides in a subset of so called peptidergic neurons. Neuropeptides are key components of the information-processing machinery of pain perception and neuropathic pain generation. Here, we compared the ability of CAPS1 and CAPS2 to support priming of both vesicle types in single and double knock-out mouse (DRG) neurons using a variety of high-resolution live cell imaging methods. While CAPS1 was localized to synapses of all DRG neurons and promoted synaptic transmission, CAPS2 was found exclusively in peptidergic neurons and mediated LDCV exocytosis. Intriguingly, ectopic expression of CAPS2 empowered non-peptidergic neurons to drive LDCV fusion, thereby identifying CAPS2 as an essential molecular determinant for peptidergic signaling. Our results reveal that these distinct functions of both CAPS paralogs are based on their differential subcellular localization in DRG neurons. Our data suggest a major role for CAPS2 in neuropathic pain via control of neuropeptide release.

## Introduction

The calcium-dependent activator protein for secretion (CAPS) is firmly established as a priming factor for vesicles undergoing regulated exocytosis (Stevens and Rettig, 2009; James and Martin, 2013). Once thought to be exclusively involved in the exocytosis of large dense-core vesicles (LDCVs), more recent evidence supports an additional role of CAPS in synaptic vesicle (SV) priming. Two mammalian CAPS paralogs (CAPS1 and CAPS2) are the products of the *CADPS1* and *CADPS2* genes and are expressed in a developmental and tissue-specific manner (Speidel et al., 2003; Sadakata et al., 2006, 2007). In adrenal chromaffin cells, both paralogs are co-expressed and promote priming of LDCVs, thereby facilitating catecholamine release (Liu et al., 2008; Speidel et al., 2008). In the central nervous system, most neurons express only one CAPS paralog (Speidel et al., 2003; Sadakata et al., 2006). For example, excitatory hippocampal neurons predominantly express CAPS1; its loss reduces spontaneous and evoked synaptic transmission (Jockusch et al., 2007) and decreases LDCV exocytosis (Farina et al., 2015; Eckenstaler et al., 2016). In contrast, cerebellar granule cells and hippocampal inhibitory interneurons predominantly express CAPS2, which is required for LDCV exocytosis, but not for synaptic transmission (Sadakata et al., 2004; Shinoda et al., 2011). Thus, the function of CAPS paralogs appears to differ in discrete neuronal populations, possibly reflecting a differential role for both CAPS paralogs in LDCV and SV exocytosis.

Dorsal root ganglion (DRG) neurons are well-suited to investigate the potential functional differences between the CAPS paralogs due to their unique properties. Although highly diverse with regard to function, DRG neurons can be subdivided into unmyelinated non-peptidergic neurons and myelinated peptidergic neurons. While both neuron types utilize glutamate for rapid synaptic transmission, peptidergic neurons produce a wide variety of neuropeptides, such as substance P (SP), calcitonin gene-related peptide (CGRP) and neuropeptide Y (NPY; Schoenen et al., 1989). Neuropeptides are contained in LDCVs, which undergo exocytosis only upon strong stimulation (Bost et al., 2017). These peptides modulate synaptic transmission (Bird et al., 2006), alter the excitability of neurons (Abdulla et al., 2001; Sapunar et al., 2005), and participate in the generation of chronic pain (Pezet and McMahon, 2006). It was previously demonstrated that CAPS1 is expressed in all DRG neurons, while CAPS2 expression is restricted to an as yet undefined neuronal subset (Sadakata et al., 2006). In light of the apparent role of CAPS2 in LDCV release in neurons, CAPS2 expression is hypothesized to be specific to peptidergic DRG neurons. Hence, the functional differences of both CAPS paralogs may be studied in a “competitive” situation in a population of DRG neurons that co-express CAPS1 and CAPS2 and engage in synaptic transmission as well as LDCV release.

In this study, we compared LDCV and SV exocytosis in DRG neurons derived from wild-type (WT), CAPS1-deficient (CAPS1 KO), CAPS2-deficient (CAPS2 KO), and CAPS1/CAPS2 double-deficient (CAPS DKO) mice (Speidel et al., 2003; Jockusch et al., 2007), correlating the functional deficits with the expression patterns of both CAPS paralogs. We demonstrate that CAPS1 is expressed in all DRG neurons, while CAPS2 is found almost exclusively in peptidergic neurons. We further demonstrate for the first time that CAPS1 and CAPS2 differentially promote SV and LDCV priming in WT DRG neurons. Our experiments also revealed that ectopic expression of CAPS2 in non-peptidergic neurons converts them to peptidergic-like neurons, and that CAPS2 plays an indirect role in synaptic transmission via neuropeptide release. Because neuropeptides significantly shape nociception (Hoyer and Bartfai, 2012), our findings imply that CAPS2-mediated peptide release plays a major role in pain sensation and in the generation of chronic pain, thus identifying this protein as an interesting novel target for the therapeutic treatment of chronic pain conditions.

## Materials and Methods

### Ethical Considerations

Procedures involving mice complied with the ethical guidelines for the care and use of laboratory animals issued by the German Government and were approved by the Institutional Animal Care and Use Committees at Saarland University, Saarland, Germany. Mice were maintained in a pathogen-free facility under standard housing conditions on a diurnal 12-h light/dark cycle with continuous access to food and water.

### Cell Culture and Transfection of DRG Neurons, isolectin B_4_ (iB_4_) Staining

Jung adult (1.5–3 week-old) WT and CAPS2 KO mouse DRG neuron cultures were generated as described previously (Bost et al., 2017). CAPS1 KO, CAPS DKO and WT control DRG neurons were isolated from E17 to E18 embryos and subjected to short enzymatic treatment for 2.5–3 min while DRGs isolated from adult mice were treated for 17 min with the enzymes. CAPS1 KO, CAPS2 KO and CAPS DKO genotypes were verified by PCR using primers as described previously (Speidel et al., 2005; Jockusch et al., 2007). Some life cell experiments required that we identify peptidergic and non-peptidergic DRG neurons. Typically, peptidergic neurons are characterized by their expression of CGRP and SP (SP/TAC1), but expression of other peptides such as NPY, galanin, or VIP can result in confounding findings (Schoenen et al., 1989). Thus, two other staining methods are widely used to identify these two populations of neurons. These methods are based on the expression of neurofilament 200 (NF200) and binding of dye-coupled isolectin B_4_ (iB_4_; Stucky and Lewin, 1999; Bae et al., 2015). Although recent single cell RNA sequencing analysis showed that classification of DRG neurons needs to be more differentiated they also showed that these methods remain partially valid (Usoskin et al., 2015; Li et al., 2016). Usoskin et al. (2015) found that NF200 is not a good marker for peptidergic neurons as non peptidergic neurons and only one subtype of peptidergic neurons contained NF200 RNA. In contrast Li et al. (2016) found that NF200 is correctly expressed by one subtype of large peptidergic DRG neurons. However, similar to our results (Supplementary Figure S1B) they also showed that some non-peptidergic neurons expressed it. The alternative staining method is iB_4_, which is mainly used to identify non-peptidergic cells. We found that about 11% of iB_4_ positive cells express SP (Supplementary Figure S1A) and Li et al. (2016) showed that about 30% of iB_4_ positive cells were effectively peptidergic. Nevertheless, in WT mice, iB_4_ labeling remains the only method that can be used with living cells; hence we primarily used this compound to identify non-peptidergic cells. We applied 2.2 μg·ml^−1^ iB_4_ in culture medium and incubated the cells 20 min at 37°C. We washed the cells twice with fresh extracellular medium before selecting the cells for life cell experiments.

### Co-culture of DRG and Spinal Cord Neurons

Embryonic E17-E18 DRG neurons were prepared from WT, CAPS1 KO, CAPS2 KO, or CAPS DKO mice as described above. Neurons that were used to measure synaptic transmission were transfected on day *in vitro* (DIV) 1 with the lentivirus encoding for Synaptophysin-pHluorin (SypHy, see below “Cell Transfection” Section). The following day, the lentivirus was removed by washing before seeding the second order spinal cord (SC) interneurons (SC neurons). This infection protocol ensured exclusive transfection of DRG neurons. The SC neurons were prepared from WT P0-P1 pups using a modified method described previously (Jo et al., 1998). Briefly, the SC was isolated, and digested for 30 min with papain (15 U/ml; Worthington Biochemical Corporation) in 5 ml of oxygenated divalent-free DMEM (Fisher Scientifics) containing 0.5 mM EDTA (Sigma) and 1 mM of CaCl_2_ and 1.64 of mM L-Cystein (Sigma). The enzymatic digestion was halted with 1 ml of culture medium (see below) and a mechanical dissociation was performed. The suspension was centrifuged for 5 min at 100× *g*. The supernatant was discarded and replaced by 500 μl EBSS (with Ca^2+^ and Mg^2+^) containing bovine serum albumin (BSA; 1 mg/ml; Sigma), trypsin inhibitor (1 mg/ml; Sigma) and DNase (1 mg/ml, Sigma). The cells were triturated gently and added to 1 ml of EBSS containing 10 mg/ml BSA and 10 mg/ml trypsin inhibitor. This step was repeated three times. After 5 min centrifugation at 100× *g*, the supernatant was replaced by 500 μl of culture medium containing Neurobasal A (Fisher Scientifics), fetal calf serum (5% v/v; Fisher Scientifics), heat-inactivated horse serum (5% v/v; Pan Biotech), penicillin and streptomycin (0.2% each; Fisher Scientifics), B27 supplement (2%, Fisher Scientifics), and β-nerve growth factor (2 μl/ml, Alomone labs). The cells were re-suspended and the supernatant was transferred to a new 15 ml tube. This step was repeated three times. The cell suspension was centrifuged at 100× *g* for 5 min, the supernatant was discarded, and the cells were re-suspended in 600 μl of culture medium and seeded on the DRG neurons. The following day, two thirds of the medium was replaced with fresh medium containing 0.28 mM of 5-fluorodeoxyuridine (Sigma). Synaptic transmission was studied DIV 8 after seeding the SC neurons.

Because most former studies using DRG/SC neuron co-cultures were performed on cells isolated from rats rather than mice, we examined synapse formations in a culture of DRG neurons isolated from synaptobrevin2-mRFP knock-in mice (SybKI; Matti et al., 2013) and SC neurons from WT animals (Supplementary Figure S2). This allowed for the distinction between synapses connecting DRG and SC neurons and synapses amid SC neurons. We then stained these neurons with antibodies against bassoon as a presynaptic marker and postsynaptic density protein 95 as a postsynaptic marker. We found that DIV 8 was best to measure synaptic transmission because at that time point the number of mature synapses reached maximum and the number of viable DRG and SC neurons was still acceptable (Supplementary Figure S2B). We also found that DRG/SC neuron synaptic contacts were located significantly closer to SC neurons than to DRG neurons, thereby reproducing the situation found *in vivo* (Supplementary Figure S2D). This result allowed us to define the optimal field of view for measuring synaptic transmission.

### Cell Transfection

To label LDCVs or SVs in DRG neurons we used the Lentivirus transfection system that allows long term expression of the gene product. Infection occurred on DIV 1 in the absence of penicillin/streptomycin. The lentivirus was removed on DIV 2 by washing with culture medium containing 0.4% penicillin and streptomycin (Fisher Scientifics). LDCVs were labeled through ectopic expression of NPY-Venus (Bost et al., 2017). The lentivirus encoding for NPY-Venus infected all neurons, irrespective of their peptidergic subtype, and induced expression of NPY-Venus, that was correctly located in LDCVs (Supplementary Figure S3). Synaptic transmission was measured with a pHluorin-based imaging method, in which super-ecliptic pHluorin is attached to synaptophysin, ensuring its exclusive localization to SVs (Miesenböck et al., 1998; Granseth et al., 2006). This Lentivirus encoding for SypHy was purchased at the Viral-Core-Facility of the Charité-Universitätsmedizin Berlin Germany (Herman et al., 2014).

For rescue experiments in which only short term overexpression of CAPS was required we used the Semliki Forest Virus transfection system. Full-length murine CAPS1 or CAPS2b, were cloned into a Semliki Forest Virus expression vector (pSFV1) containing an Internal Ribosomal Entry Site (IRES) followed by mTFP. The initial vectors CAPS1-IRES-green fluorescent protein (GFP) and CAPS2b-IRES-GFP were described previously (Liu et al., 2008). GFP was replaced with mTFP using ClaI and SpeI restriction enzymes involving a triple fragments ligation strategy. The incubation time with the virus was 5.5 h, which led to a three-fold increase in the level of CAPS1 or CAPS2 in comparison to their respective concentration in non-infected WT control cells (Supplementary Figure S4). mTFP fluorescence intensity allowed for identification of the cells that were transfected with the construct and expressed CAPS1 or CAPS2 at a controlled level. Recordings were performed within the subsequent 50 min at 32°C.

### Western Blot

Samples were collected from E18 embryos and P7 pups, and 11 μg of each sample was diluted with 5× sample buffer and homogenization buffer. Then samples were boiled for 5 min at 95°C and loaded onto an 8% SDS-polyacrylamide gel. The gels were run for 20 min at 100 V and then for 50 min at 160 V. They were then blotted onto nitrocellulose membranes (wet blot 320 mA for 2 h) and blocked using 5% milk in tris-buffered saline with Tween 20 (TBST) for 1 h. The membranes were incubated with primary antibodies against either CAPS1 (1:500) or CAPS2 (1:3,000; Table 1) diluted in TBST with 1% milk overnight. The membranes were washed three times the following day with 1% milk with TBST for 15 min. The secondary goat anti-rabbit antibody was used for both primary antibodies at a dilution of 1:3,000 in TBST with 1% milk. Following 1 h incubation, the membranes were washed with TBST three times for 15 min. They were then incubated 5 min in an enhanced chemiluminescence detection reagent and detected by the FluorChem M system (Protein Simple). β-actin labeling was also performed to correct for the loading of samples using a primary antibody (1:5,000) and a secondary goat anti-mouse antibody (1:1,000). Quantification of CAPS1 and CAPS2 protein levels in the western blot (WB) were based on β-actin as loading control and normalized to the protein expression level at P7.

**Table 1:** Details of antibodies used in the study.

Antibody	Host	Immunogen	Manufacturer and catalog no.	Working dilution
**Primary antibody**				
CAPS1	Rabbit	Recombinant protein (aa 18–107 of mouse CAPS1)	Synaptic systems (No. 262 013)	1:1,000 and 1:500
CAPS2	Rabbit	Full length CAPS2e. This antibody was immuno-purified against a CAPS2 specific sequence GSGGGAARPV	Provided by M. Jung	1:1,500
Synapsin	Guinea pig	Synthetic peptide: aa 2–28 (rat)	Synaptic systems (No. 106 004)	1:1,000
Bassoon	Rabbit	Recombinant protein aa 330—C terminal (rat)	Synaptic systems (No. 141 003)	1:300
PSD95 (monoclonal)	Mouse	Fusion protein aa 77-299 (human)	NeuroMab (Clone K28/43)	1:500
eGFP	Rabbit	Full length protein	Life technologies (G10362)	1:20
Chromogranin A	Rabbit	Recombinant fragment from the C-terminal (human)	Abcam (ab15160)	1:1,000
NF200	Mouse	C-terminal tail segment of dephosphorylated NF200 (H) subunit	Sigma Aldrich (N0142)	1:5,000
Substance P/TAC1	Rabbit	Human TAC1-GST fusion protein (Ag4790)	Proteintech (13839-1-AP)	1:200
β-actin	Mouse	Monoclonal, clone AC-15	Sigma Aldrich (A1978)	1:5,000
**Secondary antibody**		**Life technologies, Invitrogen**		
Alexa 488		Goat anti-mouse	A-11001	1:2,000
Alexa 488		Goat anti-guinea pig	A-11073	1:2,000
Alexa 568		Goat anti-mouse	A-11004	1:2,000
Alexa 647		Goat anti-mouse	A-21235	1:2,000
Alexa 568		Goat anti-rabbit	A-11011	1:2,000
Alexa 488		Goat anti-rabbit	A-11008	1:2,000
Alexa 647		Goat anti-rabbit	A-21244	1:2,000
Fab fragments		Goat anti-mouse (IgG H&L)	Rockland inc. (810–1102)	1:50
STAR Red		Goat anti-rabbit (IgG)	Abberior (2-0012-011-9)	1:100

### Immunochemistry and Confocal Imaging

Neurons were fixed at 22 ± 2°C with 4% paraformaldehyde in PBS, pH 7.4, for 10 or 20 min and then permeabilized with 0.1% Triton X-100 and 2.5% normal goat serum (NGS) in PBS for 30 min in the presence of iT-FX image enhancer (Life Technologies). The samples were blocked for 15 min using 2.5% NGS in PBS and then incubated 1 h with the indicated primary antibodies (Table 1). After several washing steps, the samples were incubated for 45 min with secondary antibodies (Invitrogen) at a 1:2,000 or 1:1,000 dilutions. If required, this was also the stage at which labeling with iB_4_ at 2 μg·ml^−1^ was performed. For immunostainings that included two primary antibodies raised in the same species, an intermediate blocking step involving Fab fragments (Rockland) was applied for 1 h and followed by extensive washing steps. Samples were mounted with home-made Mowiol-based mounting medium and imaged within 24 h of fixation with a confocal laser scanning microscope LSM 780 (Carl Zeiss) using a 63×/1.4 NA oil immersion objective. The specificity of anti-CAPS1 and anti-CAPS2 antibody was verified comparing the signal in WT neurons with DKO or single CAPS KO DRG neurons (Supplementary Figure S5).

### Total Internal Reflection Fluorescence Microscopy Imaging

The imaging setup was the same as described previously (Bost et al., 2017). It was based on an Olympus IX70 microscope equipped with a 100×/1.45 NA Plan Apochromat Olympus objective, a TILL-total internal reflection fluorescence (TILL-TIRF) condenser (TILL Photonics, Gräfelfing, Germany), and a QuantEM 512SC camera (Photometrics). It also included two lasers: a multi-band argon laser (Spectrophysics) emitting at 450, 488 and 514 nm and a solid-state laser 85 YCA emitting at 561 nm (Melles Griot). For multicolor imaging, we used a dual-view camera splitter (Visitron, Puchheim, Germany) with a cut-off of 590 ± 10 nm, separating the red and green channels. Neurons were grown on 25 mm high-precision glass coverslips (No. 1.5H, Marienfeld) to allow for even TIRF illumination.

Secretion was evoked by electrical stimulation via a bipolar platinum-iridium field electrode (#PI2ST30.5B10, MicroProbes) and a pulse stimulator (Isolated Pulse Stimulator Model 2100, A-M Systems). The settings were 4 V at 100 Hz or 10 Hz to elicit exocytosis of LDCVs and SVs, respectively. Both evoked robust [Ca^2+^]_i_ increase as assessed by Fura2 imaging (Supplementary Figure S6). LDCV secretion from NPY-Venus-expressing neurons was detected at the cell soma at 514 nm in TIRF mode. Synaptic transmission was measured in SypHy-expressing neurons that were monitored using a 488-nm laser in epi-fluorescence mode. The perfusion system comprised an in-line solution heater, which maintained the bath temperature at 32°C (Warner Instruments). The extracellular solution contained (in mM) 147 NaCl, 2.4 KCl, 2.5 CaCl_2_, 1.2 MgCl_2_, 10 HEPES and 10 Glucose, pH: 7.4 (~300 mOsm). The NH_4_Cl solution was of the same composition as the extracellular solution, but 40 mM NaCl was replaced by 40 mM NH_4_Cl. To inhibit auto- or paracrine activation of neurons through neuropetides, we used L-703,606 (Sigma), cyclotraxin B (Tocris), and olcegepant (MedChem Express), which are antagonists of neurokinin 1 (NK1), tropomyosin receptor kinase B (TRKB), and calcitonin receptor-like receptor (CLR), respectively. Cells were incubated with 10 μM of L-703,606 and cyclotraxin B 2 h before the experiments to ensure full inhibition (Cazorla et al., 2010). Ten nanometer of olcegepant was applied to the cells only 15 min before the experiment to limit the known toxic side effects (Nitzan-Luques et al., 2013). Olcegepant stock solution contained dimethyl sulfoxide (DMSO). The solvent control measurement of SV exocytosis did not differ from the control measurement without DMSO (data not shown).

### Calcium Imaging

DRG neurons were loaded with 2 μM Fura-2 AM (ThermoFisher Scientific) for 15 min at 37°C in culture medium and then washed once with extracellular solution. During recording, cells were continuously superfused with extracellular solution at 32°C. Recording was performed on the described TIRF-Microscope, which was also equipped with a VisiChrome Polychromatic Illumination System (Visitron, Puchheim, Germany) and a 40× UAPO/340 Objective (Olympus). Fura2 fluorescence was excited with 1 Hz frequency at 350 and 380 nm and emission images were collected at ≥510 nm.

### STED Microscopy

Imaging was performed with a four color STED QuadScan (Abberior Instruments GmbH) using the 640 nm excitation pulsed laser line set to 10% intensity and 775 nm STED laser to visualize LDCVs. Nominal STED laser power was set to ~20% of the maximal power of 1250 mW (corresponding to 30–34 mW in the focus, repetition rate 40 MHz) and a gating of 780 ps. Pixel dwell time and size was 10 μs and 25 nm with line accumulation set to 2. The acquisition protocol was the following: first five axial sections in confocal mode were recorded at 561 nm to visualize the iB_4_ staining, then a single confocal followed by a STED section of the cells was recorded at 647 nm to visualize the LDCVs.

### Image Analysis

LDCV exocytosis was analyzed using ImageJ v1.49i to v1.51t^1^. It was identified by rapid (within 200 ms) disappearance of the vesicle, or as a short transient increase in its fluorescence intensity accompanied by a cloud of the released NPY-Venus. The later form of exocytosis was identified as kiss and run and was detected due to NPY-Venus pH sensitivity. For more details see Bost et al. (2017). In the graphs presenting cumulative LDCV exocytosis and the density of LDCV at the footprint of the neurons, we normalized the number of LDCVs to the surface area of the cell’s footprint and multiplied it by the average area of all cells footprint in the entire graph independent of the genotype (Hugo et al., 2013). The footprint of a neuron corresponds to the membrane at the soma that is close to the glass/cell interface. Else, the raw data are presented.

SypHy data were also analyzed using ImageJ. Synapses were identified by several criteria. They corresponded to immobile stained puncta responding to 40 mM NH_4_Cl application with a sharp increase in fluorescence intensity. Synapses were marked by regions of interest (ROIs) and the mean gray value was measured as a function of time after background subtraction. The expression level of SypHy varied between cells, resulting in a variable amount of SypHy per SV, and thereby affecting the extent by which the fluorescence intensity changed upon stimulation. To overcome this problem, we neutralized the lumina of all SVs by briefly applying 40 mM NH_4_^+^ at the end of each experiment, thus allowing for the visualization of the total SypHy pool at each synapse (Sankaranarayanan and Ryan, 2000). The maximum fluorescence intensity upon NH_4_Cl application was then used to normalize the SypHy signal. Finally, to measure the time between stimulus onset and synaptic activity, we determined the time point at which the fluorescence intensity of the synapses increased by a factor of two compared to the standard deviation of the fluorescence intensity fluctuation.

Immunocytochemistry data were analyzed using ImageJ. The background was subtracted and the mean gray value was quantified. To perform CAPS1, Synaptobrevin2-mRFP (Syb2-mRFP) and synapsin co-localization analysis, we acquired confocal stacks of six to eight slices with 0.5 μm interval. Each field of view was 225 × 225 μm and the voxel size was 220 × 220 × 500 nm. Single-plane images were analyzed in two different manners: (1) Manders’ coefficient was measured using the JACoP plugin (Bolte and Cordelières, 2006) in ImageJ. This co-localization analysis was restricted to neurites of DRG neurons that were over 20 μm in length, which were characterized by their Syb2-mRFP fluorescence and were in contact with SC neurons; and (2) Line profile analysis was performed on heterotypic synapses. These synapses were identified independently of CAPS fluorescence signals. In fact, the identification was based on co-localization of Syb2-mRFP and anti-synapsin fluorescent puncta whose respective fluorescent intensities was 1,000 AU higher than the fluorescence intensities of adjacent neurites. The profiles were measured on three pixel-wide and approximatively 4 μm-long lines crossing the synapse along the neurite. The center of the synapse was defined by the Syb2-mRFP maximum fluorescence intensity. This position on the line was used to align the individual line profiles of each synapse prior to averaging.

For analyzing the synapse formations between DRG and spinal neurons (Supplementary Figure S2), synapses were marked by ROIs and counted via in house-written macro for ImageJ. The number of synapses was normalized to the surface area of the image. Using another in house-written macro, the distances between individual synapses and the plasma membrane of the nearest SC neuron soma were measured (Supplementary Figure S2C). When we measured the distances between synapses and DRG neurons, we first attempted to identify in which DRG neuron the synapse was located by tracing its neurite to the cell soma. We then measured the linear distance between the plasma membrane of this DRG neuron soma and the synapse. If backtracking was not possible due to a dense neurite network, we measured the linear distance between the synapse and the two nearest DRG neuron soma.

The size of individual LDCVs visualized with STED microscopy (Supplementary Figures S3C,D) was measured as follows. Individual vesicle images were cut out of the image of the cell (top picture), imported in Igor (Wavemetrics) and fitted by equation 1 where *A* is the amplitude, *cor* the cross-correlation term that lays between −1 and 1, *x*_0_, *y*_0_ are the center coordinates and *x*_width_, *y*_width_ are the *x* and *y* half width at half maximum of the vesicle.

(1) $$f(x)=A\cdot \exp\left[\frac{-1}{2(1-cor^{2})}\left({\left(\frac{x-x_{0}}{x_{\text{width}}}\right)}^{2}+{\left(\frac{y-y_{0}}{y_{\text{width}}}\right)}^{2}-\frac{2\cdot cor\cdot (x-x_{0})\cdot (y-y_{0})}{x_{\text{width}}\cdot y_{\text{width}}}\right)\right]$$

For calcium imaging analysis (Supplementary Figure S6), the images were background subtracted and fluorescence intensity of cells was measured using ImageJ. The ratio of F350/F380 was converted to approximate [Ca^2+^]_i_ as described by Grynkiewicz et al. (1985). *In situ* calibration was performed on cells patch-clamped in whole-cell mode with pipettes containing various Ca-EGTA buffers together with 0.1 μM Fura-2. R_(350/380)_max was 3.73, R_(350/380)_min was 0.64, Kd was 350 nM and β_(380min/380max)_ was 6.73.

### Statistics

Error bars are displayed as a standard error of the mean, and were calculated using Excel or SigmaPlot 13. The Mann-Whitney test, *t*-test, one-way ANOVA, and two-way ANOVA were calculated using SigmaPlot 13. A *p* value <0.05 was considered to be significant. The majority of the displayed graphs were generated by SigmaPlot and the remaining graphs were generated by Igor or Excel. The graphs and models were formatted primarily with CorelDRAW X6 and partially with Adobe Photoshop Cs5.

## Results

### CAPS Paralogs Are Differentially Expressed in DRG Neurons in a Time-Dependent Manner

**Figure 1:**
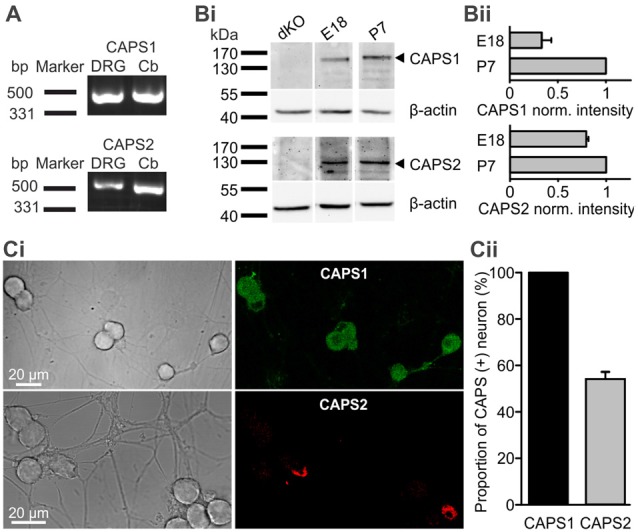
All dorsal root ganglion (DRG) neurons express calcium-dependent activator protein for secretion 1 (CAPS1) while only 50% express CAPS2. **(A)** CAPS1 and, to a lesser extent, CAPS2 mRNA was detected in DRGs. Total RNA from the DRGs (five adult mice) and cerebellum as positive control was probed for CAPS1 and CAPS2 by RT-PCR. **(Bi)** Western blot (WB) analysis of CAPS protein expression during development. DRGs were isolated from wild-type (WT) and CAPS DKO E18, and WT P7 mice and blotted with the indicated antibodies (Table 1). Actin was used as a loading control. **(Bii)** Actin-based corrected protein expression levels were normalized to the P7 signal (*n* = 2).** (Ci)** Immunocytochemistry of DRG neurons in culture with anti-CAPS1 (top) and anti-CAPS2 (bottom) antibody suggests that CAPS1 is present in all DRG neurons while CAPS2 is present in a subpopulation of DRG neurons. The specificity of the antibodies was verified (Supplementary Figure S5). **(Cii)** Quantification of CAPS paralog expression among DRG neurons (*N*_mice_ = 5, *n*_neuron_ = 70 and 191 for CAPS1 and CAPS2, respectively).

We first analyzed expression of CAPS1 and CAPS2 paralogs over time. Using RT-PCR we found that both CAPS paralogs were present at the mRNA level in DRGs of 2–3 week-old mice (Figure 1A). WB analysis of DRG homogenates revealed that both paralogs were expressed on E18. While the CAPS2 level remained relatively constant until P7, CAPS1 expression increased more than two-fold in the first week after birth (Figures 1Bi,ii), which is comparable to the expression level of the CAPS paralogs in the brain (Speidel et al., 2003). Because the homogenates contained not only neurons but also glia and other cell types, we applied immunocytochemistry to verify that the WB analysis was representative of neuronal CAPS expression. The same antibodies as in the WBs were applied to 2-week-old DRG culture maintained for 7 DIV. The specificity was tested using CAPS DKO neurons, as well as CAPS1 and CAPS2 KO neurons, to exclude cross-reactivity of antibodies (Supplementary Figure S5). The images of DRG neurons in culture that were labeled with either anti-CAPS1 or anti-CAPS2 antibodies revealed that the glial cells were almost devoid of staining (Figure 1Ci), indicating that the WB analysis reliably reported CAPS expression in neurons. More importantly, immunocytochemistry demonstrated that all DRG neurons expressed CAPS1 while only 53.7 ± 3.1% expressed CAPS2 (Figure 1Cii), confirming previous findings (Sadakata et al., 2006). The differential expression patterns of these paralogs might indicate different functions in regulating LDCV release and synaptic transmission.

### Both CAPS Paralogs Can Promote Exocytosis of LDCVs

**Figure 2:**
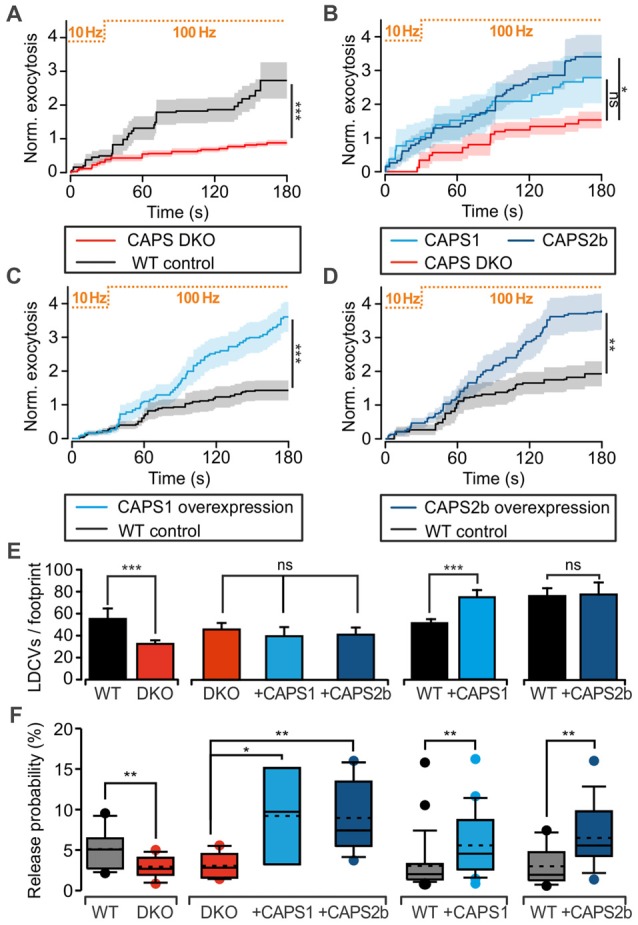
CAPS promotes large dense-core vesicle (LDCV) secretion in DRG neurons.** (A–D)** Measurement of LDCV exocytosis. LDCVs were labeled via neuropeptide Y (NPY)-Venus overexpression and visualized using TIRFM at 10 Hz. Their exocytosis was evoked via field electrode stimulation. The protocol consisting of a 30-s pre-stimulus at 10 Hz followed by a 100 Hz stimulus is illustrated at the top of each graph. LDCV exocytosis is displayed as the average cumulative exocytosis normalized to the cell footprint area. The graphs include only the cells that responded to the stimulus with LDCV exocytosis. Error bars are SEM. **(A)** CAPS DKO E18 DRG neurons exhibited defective LDCV secretion compared to E18 WT neurons (N_pups_ = 5 for WT and 6 for CAPS DKO, n_neurons_ = 16 and 18 for WT and CAPS DKO, respectively). **(B)** Overexpression of CAPS2 rescued LDCV secretion in E18 CAPS DKO neurons (N_pups_ = 10 E18 embryos, n_neurons_ = 10, 8, and 12 for CAPS DKO, CAPS DKO expressing CAPS1, and CAPS DKO expressing CAPS2, respectively). **(C)** CAPS1 overexpression in adult WT neurons strongly enhanced LDCV secretion (N_mice_ = 5, n_neurons_ = 26 and 23 for WT and WT overexpressing CAPS1, respectively). **(D)** CAPS2 overexpression in adult WT neurons induced a robust increase in LDCV secretion (N_mice_ = 7, n_neurons_ = 18 and 15 for WT and WT transfected with CAPS2, respectively). Note that secretion in adult WT neurons (panels **C,D**) was lower than that in E18 WT neurons (panel **A**). **(E)** Average number of LDCVs visible in the evanescent field normalized to the cell footprint area. Error bars are SEM. **(F)** Box plot of LDCV release probability, which was calculated by dividing the number of fusion events by the total number of LDCVs at the footprint of the neurons presented in panels **(A–D)**. The stippled and solid lines in the box correspond to the average and median release probability, respectively. The Mann-Whitney significance test was applied for all graphs except for **(B)** and the second plot in **(E,F)**, in which an ANOVA on ranks with Dunn’s post significance test was applied; *p < 0.05, **p < 0.01, ***p < 0.001, ns (not significant) *p* > 0.05.

We assessed CAPS function in LDCV exocytosis at the somata of 7 DIV DRG neurons with TIRFM. LDCVs were specifically labeled by NPY-Venus expression using a lentivirus (Bost et al., 2017) and exocytosis was triggered via field electrode stimulation at 100 Hz. A pre-stimulus at 10 Hz was applied to the cells for 30 s in order to elevate intracellular calcium and promote priming of LDCVs (Bost et al., 2017). In WT neurons, this pre-stimulus induced a moderate rate of LDCV secretion, which was almost doubled by 100 Hz stimulation (Figure 2). After 3 min of stimulation, the WT neurons secreted, on average, 2.7 ± 0.5 LDCVs (Figure 2A). The LDCV release probability, defined as the number of exocytosis events divided by the total number of LDCVs per footprint area (Figure 2E), was 5.1 ± 0.6% (Figure 2F), which is approximately twice as high as that in hippocampal neurons (van Keimpema et al., 2017). In CAPS DKO neurons, LDCV exocytosis was reduced by ~70% while the release probability was reduced by >40% in comparison to WT neurons (Figures 2A,F). To verify that impaired LDCV exocytosis in CAPS DKO mice was due to CAPS deletion, we performed rescue experiments by transfecting CAPS DKO neurons with either CAPS1 or CAPS2 5–6 h before the experiments using a Semliki Forest virus transfection system. The number of fusing LDCVs in CAPS DKO neurons transfected with CAPS1 or CAPS2 was approximately two-fold higher than that in control DKO DRG neurons. This increase of secretion was significant in cells transfected with CAPS2, but not in cells transfected with CAPS1 (Figure 2B). Nonetheless, the release probability was significantly increased by a factor of three in DKO cells transfected with either of the CAPS paralogs in comparison to that in DKO control cells (Figure 2F). Consequently, both CAPS paralogs were able to restore LDCV exocytosis in CAPS DKO DRG neurons to a level exceeding secretion in WT neurons, prompting us to test the effect of CAPS overexpression in WT DRG neurons. We found that LDCV exocytosis and the release probability were doubled in WT cells transfected with CAPS1 or CAPS2 compared to that in control cells (Figures 2C,D,F). Overall, these results indicate that both CAPS paralogs are able to promote LDCV exocytosis in DRG neurons and that WT DRG neurons do not express CAPS at saturating concentrations.

### Only Peptidergic DRG Neurons Express CAPS2 and Secrete LDCVs Upon Stimulation

**Figure 3:**
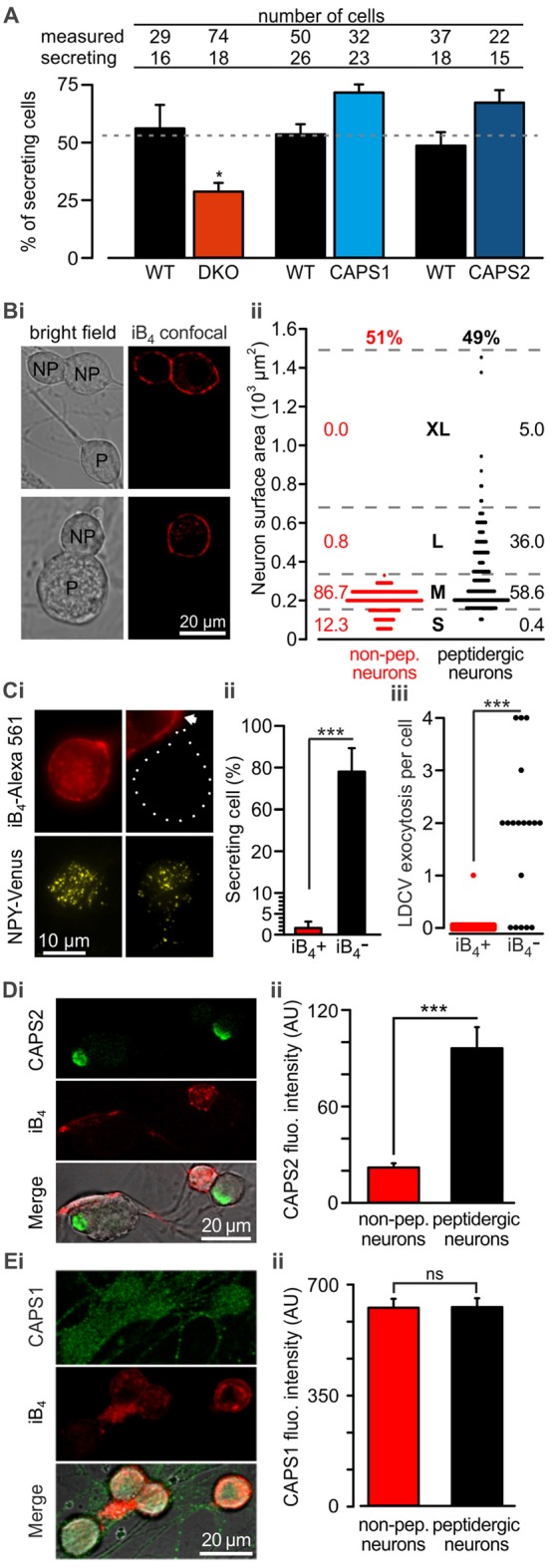
CAPS2 is localized to the secreting peptidergic neurons. **(A)** Percentage of cells that released LDCVs upon field electrode stimulation in CAPS DKO, CAPS1-, or CAPS2-overexpressing neurons compared to their WT controls. The cells that responded to the stimulus with LDCV exocytosis are presented in Figure 2. The gray dotted line corresponds to the average proportion of all responding WT controls. (N_WT & DKO_ = 5 and 6 respectively, N_CAPS1 & WT control_ = 5, N_CAPS2 & WT control_ = 7; *p < 0.05 Student *t*-test). **(Bi)** Two exemplary confocal images of live DRG neurons stained with isolectin B4 (iB4)-Alexa 561. IB_4_ binds to the plasma membrane of non-peptidergic DRG neurons but due to constitutive endocytosis some cytoplasmic staining is also visible. Stained neurons are labeled with the letters “NP” on joint bright field images. Peptidergic neurons are void of iB_4_-Alexa 561 staining and are indicated with “P” on the bright field images. **(Bii)** Scatter dot plot of peptidergic and non-peptidergic DRG neuron size. Neurons were classified as small (S), medium (M), large (L), and extra-large (XL). The thresholds between sizes (gray stippled lines) were set using a distribution histogram. Percentages of neurons in each size category are indicated at the side of the dot plot. Proportions of peptidergic and non-peptidergic DRG neurons in randomly acquired images are indicated on top (N_mice_ = 2, n_neurons_ = 226 and 210 non-peptidergic and peptidergic neurons, respectively). **(Ci)** LDCV secretion was measured in DRG neurons stained with iB_4_-Alexa 561 (top, epifluorescence images). Left and right panels present non-peptidergic and peptidergic (dotted line) DRG neurons, respectively, transfected with NPY-Venus (bottom, TIRFM images). Localization of NPY-Venus in LDCVs in peptidergic and non-peptidergic neurons was verified (Supplementary Figure S3). Arrow indicates adjacent stained non-peptidergic neuron. **(Cii)** Percentage of peptidergic and non-peptidergic neurons that released LDCV upon electrical stimulation. The stimulation protocol was identical to that shown in Figure 2. **(Ciii)** Scatter dot plots of total LDCV secretion from all peptidergic and non-peptidergic neurons. (N_mice_ = 4, n_neurons_ = 34 and 17 for non-peptidergic and peptidergic neurons, respectively). **(Di)** Representative confocal images of WT DRG neurons immuno-labeled with anti-CAPS2 antibody (top) and co-stained with iB_4_-Alexa 561 (middle). The bottom panel presents the overlay with the bright field image. **(Dii)** The CAPS2 fluorescent intensity in peptidergic and non-peptidergic neurons revealed that CAPS2 was localized to peptidergic neurons (N_mice_ = 2, n_neurons_ = 57. **(Ei)** Representative confocal images of WT DRG neurons labeled with anti-CAPS1 antibody (top), and iB_4_ (middle) and their overlay with the bright field image (bottom). **(Eii)** Quantification of CAPS1 fluorescence intensity in peptidergic and non-peptidergic neurons shows that CAPS1 is not preferentially localized to a DRG neuron subtype. *N* = 2 P0 pups, n_neurons_ = 92. *T*-test was applied in **(A)** and **(Cii)**, and the Mann-Whitney test was applied in **(Ciii)**, **(Dii)** and **(Eii)**, ns (not significant) *p* > 0.05, ***p < 0.001.

Because we analyzed LDCV exocytosis only in cells that responded to electrical stimulation with LDCV fusion, we next examined whether deletion or overexpression of CAPS paralogs affected the proportion of responding cells. We found that under the current recording conditions, 52.8 ± 2.2% of the WT DRG neurons underwent LDCV exocytosis, confirming previous findings (Bost et al., 2017; Figure 3A). CAPS1 or CAPS2 overexpression in WT neurons increased the percentage of secreting DRG neurons by 33 and 37%, respectively in comparison to their respective WT control cells (*p* = 0.18 and 0.20 respectively, Student *t*-test; N_CAPS1 & WT control_ = 5, N_CAPS2 & WT control_ = 7). Interestingly, deletion of both CAPS paralogs reduced the percentage of responding neurons to 28.8 ± 3.8%, which is significantly less than that in WT neurons (Figure 3A). Accordingly, deletion of both CAPS paralogs had a profound effect on all aspects of stimulated LDCV exocytosis.

Lack of LDCV secretion in a subset of cells may point to functional diversity among DRG neurons, which might coincide with their classification into peptidergic and non-peptidergic subtypes. Because iB_4_ consistently identified non-peptidergic DRG neurons in mice (Supplementary Figure S1, but see Li et al., 2016) and can be used on live cells, we primarily used this compound to differentiate between DRG neuron subtypes. In our culture conditions, 49% of DRG neurons were iB_4_ negative (i.e., peptidergic) neurons, while 51% were iB_4_ positive (i.e., non-peptidergic) neurons (Figures 3Bi,ii). The lentivirus infected all neurons, irrespective of the subtype, and induced expression of NPY-Venus, that was correctly located in LDCVs (Supplementary Figure S3). Furthermore, exogenous NPY-Venus expression over 7 DIV did not alter the fundamental nature of the DRG neurons, as they retained their susceptibility to staining with iB_4_-Alexa561 (Figure 3Ci). This allowed us to specifically measure LDCV fusion in peptidergic and non-peptidergic neurons. We found that only one out of 34 iB_4_ positive neurons underwent LDCV exocytosis (Figure 3Cii). In contrast, 78 ± 11% of iB_4_ negative neurons exhibited an average of 1.8 ± 0.2 LDCVs fusing with the plasma membrane upon stimulation (Figure 3Ciii). Therefore, stimulated LDCV exocytosis in WT DRG neurons almost exclusively occurs in iB_4_ negative (i.e., peptidergic) neurons.

Because all DRG neurons expressed CAPS1 but only 45% expressed CAPS2 (Figure 1C), we next investigated whether CAPS2 is expressed only in peptidergic neurons. Co-staining of WT DRG neurons with anti-CAPS2 antibody and iB_4_-Alexa561 revealed little co-localization of both markers. Overall, only a very minor fraction of the iB_4_-positive cells expressed CAPS2 (Figures 3Di,ii). In contrast, 80% of the iB_4_-negative cells expressed CAPS2. These data suggest that stimulated LDCV exocytosis in DRG neurons might be mediated exclusively by CAPS2 and not by CAPS1.

### Among CAPS Paralogs Only CAPS2 Mediates Stimulated LDCV Secretion in WT DRG Neurons

**Figure 4:**
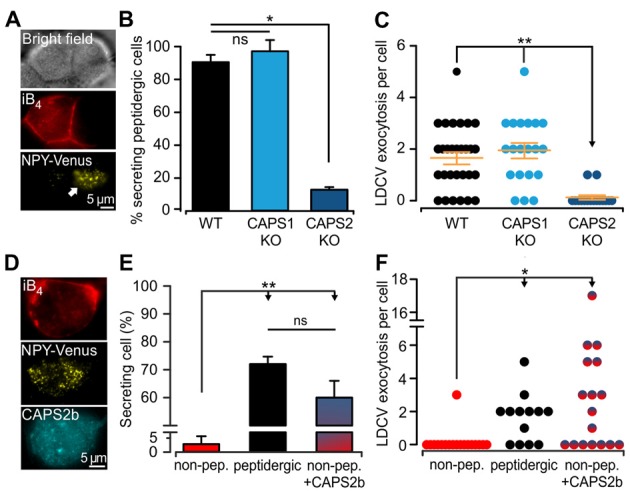
CAPS2 is the priming factor of LDCVs in DRG neurons. **(A)** Exemplary images of CAPS1 KO DRG neurons. The two cells visible in the bright field image (top) are either non-peptidergic (stained red with iB_4_-Alexa561, middle left) or peptidergic (middle right). LDCV exocytosis was measured in the NPY-Venus transfected peptidergic neuron (white arrow, total internal reflection fluorescence microscopy image, bottom). The stimulation protocol was the same as that in Figure 2. **(B)** Percentage of peptidergic neurons that secreted LDCVs depending on their genotype; which were WT, CAPS1 KO and CAPS2-deficient (CAPS2 KO). The number of preparations (i.e., mice) was 7, 7, and 4 for WT, CAPS1 KO and CAPS2 KO, respectively (ANOVA on ranks Holm-Sidak method: *p < 0.05). **(C)** Scatter dot plots of total LDCV exocytosis in the CAPS1 KO and CAPS2 KO peptidergic neurons in comparison to their respective WT controls. Orange dashes are the average LDCV exocytosis ± SEM (N_mice_ = 7, 7, and 4, n_neurons_ = 32, 20 and 16 for WT, CAPS1 KO, and CAPS2 KO, respectively; ANOVA on ranks Kruskal-Wallis test: **p < 0.01). **(D)** Representative image of a WT non-peptidergic DRG neuron co-transfected with NPY-Venus and CAPS2-mTFP. The neuron was identified by triple staining in red for iB_4_-Alexa568 (top), yellow for NPY-Venus (middle), and cyan for CAPS2-mTFP (bottom). LDCV exocytosis induced by the protocol illustrated in Figure 2 was assessed in these neurons and compared to exocytosis in WT peptidergic and non-peptidergic neurons transfected with NPY-Venus only. **(E)** Proportion of secreting WT neurons that were non-peptidergic, peptidergic and non-peptidergic transfected with CAPS2. Note that ectopic expression of CAPS2 in non-peptidergic neurons converts these neurons to LDCV secreting cells. **(F)** Scatter dot plots of total LDCV secretion from WT neurons that were non-peptidergic, peptidergic and non-peptidergic overexpressing CAPS2. For panels **(E,F)** N_mice_ = 13, n_neurons_ = 34, 13, 17 for non-peptidergic, peptidergic and non-peptidergic overexpressing CAPS2, respectively. **p < 0.01, *p < 0.05 and ns (not significant) *p* > 0.05, one-way ANOVA Dunn’s test.

The expression of CAPS2 in DRG neurons is correlated with the ability to fuse LDCVs upon stimulation. Yet, CAPS1 was expressed in all DRG neurons to the same degree (Figures 3Ei,ii), and CAPS1 overexpression in CAPS DKO neurons could restore stimulated LDCV secretion (Figure 2F). These findings prompted us to test whether CAPS1 is involved in regulated LDCV exocytosis in WT iB_4_ negative DRG neurons. We addressed this question by measuring LDCV fusion in iB_4_ negative CAPS1 KO (Figure 4A) or iB_4_ negative CAPS2 KO neurons and compared it to LDCV fusion in their WT counterparts. We found that CAPS1 deletion had no impact on LDCV exocytosis (Figures 4B,C). Conversely, the number of iB_4_ negative neurons undergoing LDCV secretion was decreased by 82% in CAPS2-deficient neurons compared to their WT controls (Figures 4B,C). These results denote that, in WT neurons, CAPS1 is not involved in peptide secretion while CAPS2 alone promotes regulated LDCV release in peptidergic (i.e., iB_4_ negative) DRG neurons.

Our data indicate that regulated LDCV exocytosis from peptidergic CAPS2 KO neurons is very similar to that of non-peptidergic WT neurons (compare Figures 4C and Figure 3C). Consequently, the absence of regulated LDCV exocytosis in non-peptidergic neurons should result from their lack of CAPS2 expression (3D). To test this hypothesis, we overexpressed CAPS2 in WT DRG neurons and studied the fusion of NPY-Venus labeled LDCVs in iB_4_-positive (i.e., non-peptidergic) neurons (Figure 4D). We found that 60 ± 6% of these neurons underwent LDCV exocytosis upon field-electrode stimulation, which is considerably higher than the very small proportion of secreting iB_4_ positive WT control neurons, but similar to that of iB_4_ negative WT control neurons (Figure 4E). The number of fusing LDCVs was slightly higher in CAPS2-transfected iB_4_ positive neurons compared to WT iB_4_ negative neurons expressing CAPS2 (Figure 4F). Overall, our results indicate that CAPS2 is the exclusive, missing key factor in non-peptidergic (i.e., iB_4_ positive) neurons that determines LDCV fusion competence. Additionally, ectopic expression of CAPS2 enables non-peptidergic neurons to secrete neuropeptides.

### CAPS1 Is Enriched at DRG/SC Neuronal Synapses

**Figure 5:**
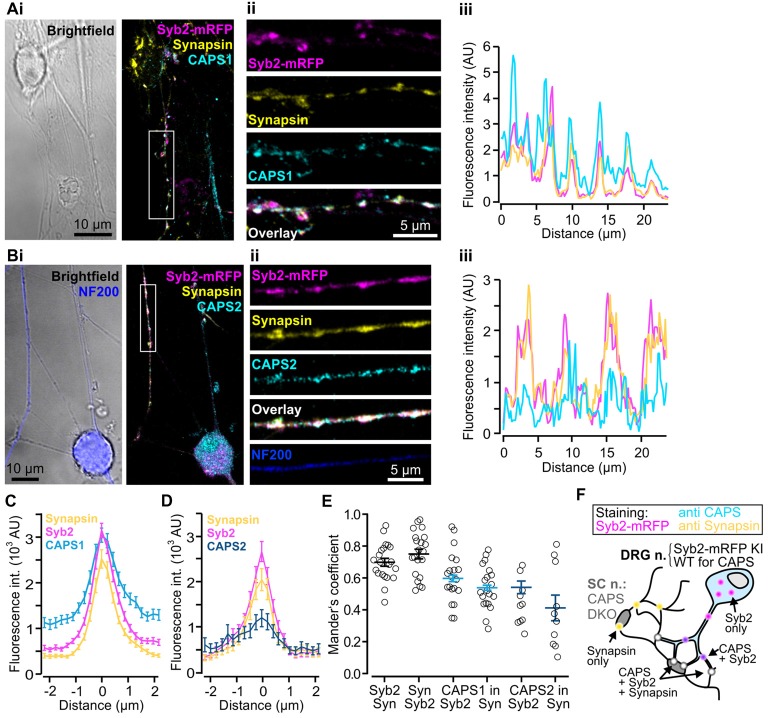
CAPS1 is localized to synapses. Co-cultured adult Synaptobrevin2-mRFP (Syb2-mRFP) knock-in DRG neurons with E18 CAPS DKO spinal cord (SC) neurons were fixed with 4% paraformaldehyde at day *in vitro* (DIV) 7 and stained with antibodies directed against CAPS1 or CAPS2 and synapsin to mark synapses. This culture condition ensured that anti-CAPS labeling was localized to DRG and not SC interneurons (SC neurons) and that DRG neurites could be readily identified by Syb2-mRFP red fluorescence. **(Ai)** Left: bright field image of one SC neuron and a network of neurites. Right: overlay of a maximal intensity projection (MIP) image of Syb2-mRFP (magenta), synapsin (yellow) and CAPS1 (cyan) labeling. **(Aii)** Enlarged portion of a neurite outlined in **(Ai)**. The individual channels (Syb2-mRFP, synapsin and CAPS1) and their overlay are displayed from top to bottom. **(Aiii)** Line profile along the neurite presented in **(Aii)**. Note that CAPS1 is enriched at synapses (puncta in which Syb2-mRFP and synapsin co-localize). **(Bi)** Left: overlay of a bright field image and anti-neurofilament 200 (NF200; blue) labeling of a peptidergic DRG neuron. Overlay of a MIP image of Syb2-mRFP (magenta), synapsin (yellow) and CAPS1 (cyan) labeling. **(Bii)** Enlarged portion of a neurite outlined in **(Bi)**. The individual channels (Syb2-mRFP, synapsin and CAPS1), their overlay, and the NF200 labeling are presented from top to bottom **(Biii)** Line profile along the neurite shown in **(Bii)**. **(C)** Average line profile diagram for CAPS1 over synapses that were selected according to co-localized high fluorescence intensities of the Syb2-mRFP and synapsin signals (*n* = 130). **(D)** Average line profile diagram over synapses for CAPS2, Syb2-mRFP and synapsin (*n* = 31). **(E)** Co-localization was tested with Manders’ coefficient measured on isolated neurites of DRG neurons. Localizations of Syb2-mRFP to synapsin and its inverse, CAPS1 to Syb2-mRFP and CAPS1 to synapsin, CAPS2 to Syb2-mRFP and CAPS2 to synapsin are presented. Averages are indicated as a black line with SEM (*n* = 21 and 10 for CAPS1 and CAPS2, respectively). **(F)** Schematic representation of the experimental design. Yellow dots indicate SC-SC neuron homotypic synapses in which, by design, only synapsin can be observed. Magenta dots likely correspond to SVs or LDCVs at extra-synaptic locations. CAPS1 was found at low levels throughout the DRG-neuron (light blue, see **(B,C)**) and enriched at heterotypic synapses (white dots) but can also co-localize with Syb2-mRFP (violet dots).

Thus far, we have demonstrated that CAPS1 is expressed at equal levels in all WT DRG neurons (Figure 3E) but that it plays no role in LDCV secretion (Figure 4B). CAPS1 has been implicated in synaptic transmission in hippocampal neurons (Jockusch et al., 2007), and we hypothesized that it might play a similar role in DRG neurons. If CAPS1 is involved in synaptic transmission in DRG neurons, it should be localized to synapses. DRG neurons do not form synapses with other DRG neurons *in vivo* or *in vitro* (Wake et al., 2015). However, *in vitro*, they can generate functional synaptic contacts with their natural target cells, which are second order SC neurons (Gu and MacDermott, 1997; Joseph et al., 2010). First, we established and optimized the mouse DRG/SC neuron co-culture (Supplementary Figure S2), and then analyzed CAPS1 and CAPS2 subcellular localization by performing immunostainings with their respective antibody. In order to identify heterotypic synapses formed between DRG and SC neurons, we isolated the DRG neurons from SybKI (Matti et al., 2013) and SC neurons from CAPS DKO mice (Figure 5F). Because, Synaptobrevin2 is mainly but not exclusively localized to synapses (Ahmari et al., 2000; Schwarz et al., 2017), we also marked synapses with anti-synapsin antibody (Chi et al., 2001). This antibody labeled heterotypic synapses and homotypic synapses among SC neurons (Figure 5F). Due to this complex staining configuration, we restricted the Manders’ coefficient analysis to DRG neurites that contacted SC neurons. This revealed a good degree of co-localization between CAPS1 and Syb2-mRFP or synapsin (0.60 ± 0.03 and 0.54 ± 0.03, *n* = 21 and 10, respectively, Figure 5E). To better assess the distribution of CAPS1 at synapses, we measured the intensity profiles of CAPS1, Syb2-mRFP and synapsin fluorescence at heterotypic synapses between DRG and SC neurons. These were readily detected as puncta in which Syb2-mRFP and synapsin were enriched and co-localized (Figures 5Ai–iii). The average intensity profiles of Syb2-mRFP and synapsin ranged from very low base line fluorescence intensities to well-defined coinciding peak fluorescence (Figures 5C,D). The CAPS1 signal was comparatively high in neurites but was markedly increased at synapses to reach a maximum that matched the Syb2-mRFP and synapsin fluorescence intensity profiles (Figure 5C). On average, the CAPS1 fluorescence intensity was 4.6 ± 0.4-folds higher at synapses than in adjacent neurites. In contrast, CAPS2 was localized along the entire neurites (Figure 5Bi–iii). Its signal at synapses increased only mildly and the peak fluorescence was poorly defined (Figure 5D). This revealed that CAPS1, but not CAPS2, is localized and enriched at synapses, thus suggesting a possible role for CAPS1 in synaptic transmission.

### CAPS1 but Not CAPS2 Promotes SV Exocytosis

**Figure 6:**
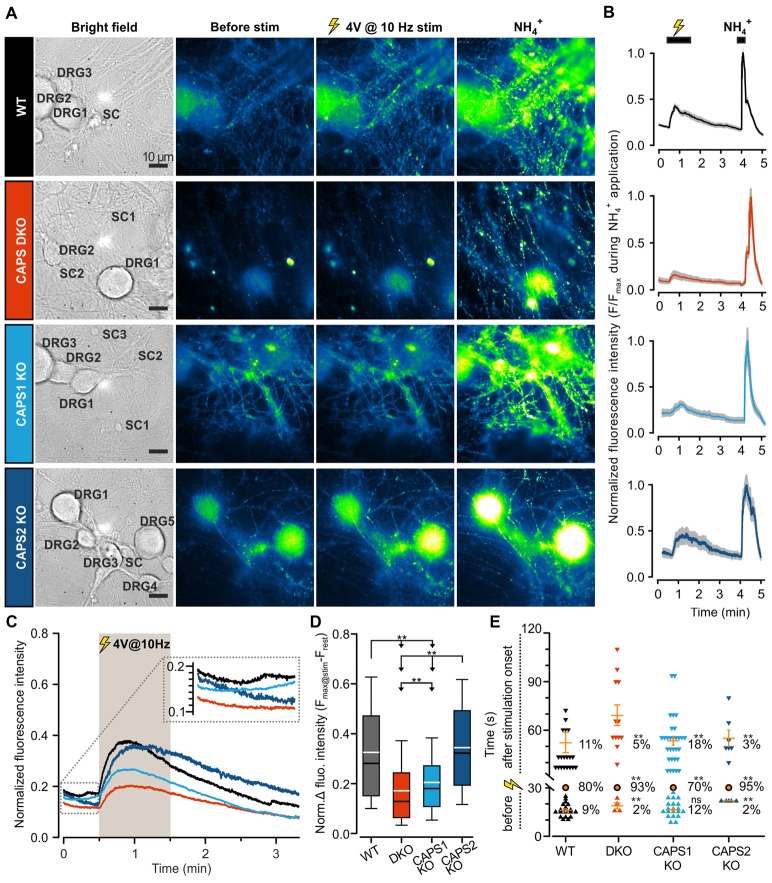
CAPS1 mediates synaptic transmission between DRG and SC neurons. Embryonic WT, CAPS DKO, CAPS1 KO or CAPS2 KO DRG neurons were transfected with Synaptophysin-pHluorin (SypHy) and co-cultured with WT SC neurons. Synaptic transmission was measured after 8 DIV. **(A)** Co-cultured neurons are shown from left to right in bright field and in epifluorescence to visualize the SypHy signal before and during 10 Hz field electrode stimulation, and upon NH_4_Cl application. For the purpose of normalization, 40 mM NH_4_Cl was used to deprotonate the lumina of synaptic vesicles (SVs), thereby allowing visualization of the entire SV pool. The neuron types are indicated in bright field images. Scale bar = 10 μm. **(B)** Time course of normalized average SypHy fluorescence intensity at synapses of the recordings displayed on the same row in **(A)**. The fluorescence intensity of each synapse was normalized to their individual maximal intensity measured during NH_4_Cl application. The SEM range is indicated by the shaded gray area. **(C)** The normalized average SypHy signal at synapses in response to electrical stimulation for WT neurons (black), CAPS DKO neurons (red), CAPS1 KO neurons (light blue) and CAPS2 KO neurons (navy blue) indicate that deletion of CAPS1 alone was sufficient to severely reduce synaptic transmission. The shaded gray area corresponds to the stimulation period. The SEMs were too small to be displayed. The inset corresponds to the fluorescence intensity fluctuation prior to stimulation. **(D)** Box plot of the maximum normalized fluorescence intensity increase in SypHy elicited by 10 Hz electrical stimulation. CAPS DKO as well as CAPS1 KO strongly reduced SV exocytosis while CAPS2 deletion had no effect. The white and black lines in the box correspond to the average and median fluorescence increase, respectively. Outliers are not displayed, in order to ease legibility. Significance was tested with ANOVA on rank and Dunn’s post significance test. **(E)** Quantification of the time point of synaptic response suggests that the presence of CAPS2 causes non-synchronized synaptic activity. The time of synaptic activity was divided into three groups. Each synapse responding either before stimulation or with a delay is represented as an individual symbol. Orange dashes are the average time points of response ± SEM for these two groups. All synapses responding within 1 s of stimulus onset are represented as a single orange circle. The percentage of synapses belonging to each group is provided to the right of response time. These percentages were tested with ANOVA on ranks and Dunn’s post significance test comparing all values to WT controls. Experiments were performed on a minimum of three independent cultures for every genotype. WT n_DRG neurons_ = 32, n_synapses_ = 209; CAPS DKO n_DRG neurons_ = 35, n_synapses_ = 252; CAPS1 KO n_DRG neurons_ = 38, n_synapses_ = 270; and CAPS2 KO n_DRG neurons =_ 28, n_synapses_ = 155. ns = not significant (*p* > 0.05), **p < 0.01.

To assess the role of both CAPS paralogs in exocytosis at synapses, we measured SV fusion using the SypHy-based imaging method. Synaptic transmission was elicited via mild field electrode stimulation at 10 Hz for 1 min and synaptic fluorescence responses were normalized to the total SV pool (maximum fluorescence measured during NH_4_^+^ application, see “Material and Methods” Section). In WT DRG neurons, synaptic activity was marked by a strong increase in the SypHy fluorescence intensity at synapses, which was substantially reduced in CAPS DKO and in CAPS1 KO neurons, but not in CAPS2 KO neurons (Figures 6A–C). Figure 6D indicates that the SypHy fluorescence intensity increase elicited by a 10 Hz depolarization train was maximal in WT neurons and reduced by a factor of two in DKO neurons. Similarly, deletion of only CAPS1 resulted in a 37% decrease in the SypHy peak response, while CAPS2 KO had no effect. Hence, in DRG neurons, synaptic transmission appears to be exclusively promoted by CAPS1 and not CAPS2.

### CAPS2 Indirectly Modulates Synaptic Transmission by Controlling Peptide Release

We noticed that the average fluorescence intensity of synapses increased slightly before stimulation in WT neurons as well as in CAPS1 KO neurons, while in CAPS2 KO and in CAPS DKO neurons, the fluorescence intensity was stable or even decreased to some extent (Figure 6C inset). We hypothesized that CAPS2 might affect the time course of synaptic activity and the time point at which individual synapses were active. Thus, we measured the delay between the stimulus and the increase in the fluorescence intensity at each synapse. Because the stimulus was triggered manually, we defined synapse synchronization as a synapse response within 1 s of stimulus onset. In WT control DRG neurons, synapse synchronization occurred in 80% of all synapses (Figure 6E). In CAPS1 KO neurons, this proportion decreased slightly to 70%, while in CAPS DKO and in CAPS2 KO neurons, more than 90% of all responding synapses were synchronized. In WT control neurons, non-synchronized release occurred to a similar extent before or after stimulus onset (9 and 11%, respectively). This finding correlates with the fact that 15% of resting DRG neurons maintained in co-culture with SC neurons exhibited spontaneous [Ca^2+^]_i_ oscillations (Supplementary Figure S6E). These oscillations were not associated with network activity because they were not inhibited by co-application of D-2-amino-5-phosphonopentanoate (APV) and 6,7-dinitroquinoxaline-2,3-dione (DNQX), which are glutamate receptor antagonists (Supplementary Figure S6F). The spontaneous synaptic transmission that occurred prior to stimulation did not differ between CAPS1 KO neurons and WT neurons. However, the percentage of asynchronous synaptic activity ensuing after the onset of stimulus was increased by 64% in CAPS1 KO neurons compared to that in WT neurons (18 and 11%, respectively, Figure 6E). In contrast, in CAPS DKO and CAPS2 KO cells, spontaneous and asynchronous responses were reduced in average by 68% compared to that in WT neurons (Figure 6E). Overall, synaptic transmission in WT and CAPS1 KO neurons was considerably less synchronized than that in CAPS DKO and CAPS2 KO neurons. We suggest that the neuropeptides that were released from CAPS2 expressing peptidergic DRG neurons (Figure 4), induced via auto- or paracrine signaling, non-synchronized synaptic activity in surrounding DRG neurons.

**Figure 7:**
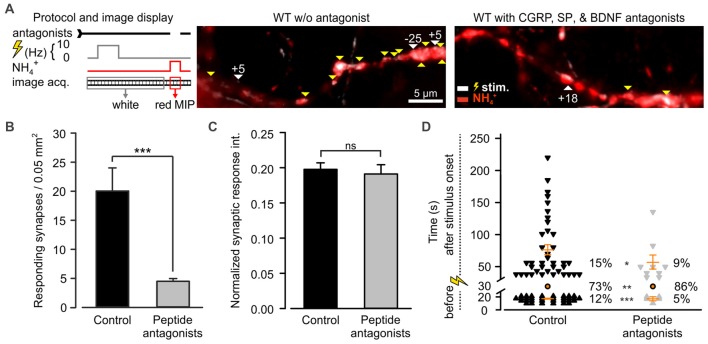
CAPS2 indirectly regulates synaptic transmission. Synaptic transmission measured in WT DRG/SC neurons co-cultures pre-incubated or not (control) with 10 nM olcegepant, 10 μM L-703,606 oxalate salt hydrate, and 10 μM cyclotraxin B, inhibiting the receptors for calcitonin gene-related peptide (CGRP) calcitonin receptor-like receptor (CLR), Substance P (SP; NK-1R), and BDNF tropomyosin receptor kinase B (TRKB), respectively. Two co-cultures were generated using two adult mice for DRG neurons and 12 P0 mice for SC neurons, N_DRG neurons_ = 29 and 50, n_synapses_ = 241 and 101 for control and treated neurons, respectively. Error bars are SEM, and Mann-Whitney test was applied, ns = non-significant, *p < 0.5, **p < 0.01 and ***p < 0.001. **(A)** The recording protocol is shown on the left. The images correspond to an overlay of the SypHy MIP over time in DRG/SC neurons acquired prior to (white) and during superfusion with NH_4_Cl (red) in control (left) and inhibition (right) conditions. White vs. red images indicate active vs. SypHy-labeled synapses, respectively. Yellow and white arrows indicate synchronized and uncoupled synapses, respectively. The associated numbers correspond to the time (s) between the stimulus and the response. **(B)** Average number of active synapses in neurons pretreated with peptide antagonist (gray) or not (black). **(C)** Average SypHy fluorescence increase in response to electrical stimulation normalized to SypHy fluorescence upon NH_4_Cl application in control and treated neurons. **(D)** Density dot plot illustrating the time point of activity and the percentage of synapses that were either synchronized to the stimulus or that responded before or after the stimulus. The time point of response of each unsynchronized synapse is indicated by individual symbols whereas all synchronized synapses are indicated by one orange circle. Orange dashes are the average time point of unsynchronized response ± SEM.

Among the most abundant pro-inflammatory peptides expressed by DRG neurons are CGRP and SP (Schoenen et al., 1989). Both have been shown to sensitize spinal dorsal horn neurons and DRG neurons (Abdulla et al., 2001; Russell et al., 2014). Consequently, they could play a role in desynchronization of synaptic transmission. These peptides were shown to co-localize in LDCVs of DRG neurons with BDNF, a major player in neuroplasticity (Salio et al., 2007). Moreover, a large number of DRG neurons express BDNF receptor TRKB, SP receptor NK1 (Lever et al., 2003; Merighi et al., 2008) and CGRP receptor (CLR; Cottrell et al., 2005). Hence, to test whether CAPS2 contributes to synaptic activity indirectly via peptide secretion, we treated DRG neurons with L-703,606, olcegepant and cyclotraxin B, which are SP, CGRP and BDNF antagonists, respectively. Figure 7A presents two exemplary recordings in which WT neurons were treated or not (control) by the antagonists. In control neurons, the vast majority of the synaptic sites were active, while in neurons treated with inhibitors, only a few synapses were active upon depolarization. Overall, approximately one fifth of the treated synapses responded to the stimulus compared to that in control neurons (Figure 7B). This marked reduction in the number of active synapses can be explained by the long-term effects of the TRKB antagonist, which inhibits BDNF-induced synaptic potentiation (Edelmann et al., 2014). With regard to the responding synapses, the change in the fluorescence intensity of SypHy was identical whether the cells were treated or not (Figure 7C). This indicates that the number of SVs released at the responding synapses was treatment independent. More importantly, exocytosis of SVs was significantly better synchronized to the stimulus upon antagonist treatment than in untreated control cells (Figure 7D). In control neurons, only 73% of the synapses exhibited a fluorescence intensity increase upon depolarization, but this percentage increased to 86% in treated neurons. The 2.4-fold reduction in spontaneous synaptic activity (i.e., prior to stimulation) in treated neurons compared to control neurons was essentially responsible for the synchronization of synaptic transmission. In summary, blocking the effects of SP, CGRP and BDNF via antagonist treatment reproduced the effect of CAPS2 deletion on non-synchronous synaptic activity. Therefore, the results indicate that while CAPS1 directly promotes SV exocytosis, CAPS2 indirectly modulates synaptic transmission via control of neuropeptide release from LDCVs.

## Discussion

**Figure 8:**
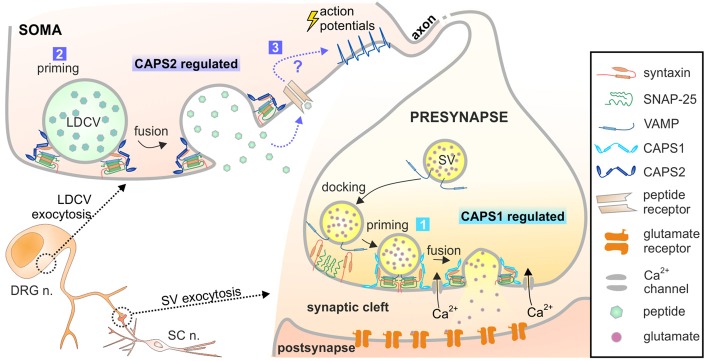
Model of CAPS1 and CAPS2 function in DRG neurons. Our data suggest that 1. SV exocytosis is promoted by CAPS1 and 2. LDCV exocytosis is mediated by CAPS2 most likely by enhancing priming. 3. Peptidergic cargo released by LDCVs binds to its associated receptor and indirectly induces SV fusion by a yet unknown mechanism. The peptides might act in an autocrine pathway as depicted here or in a paracrine pathway and thereby affect synaptic transmission of many neighboring neurons.

We investigated the role of the CAPS1 and CAPS2 paralogs in exocytosis of SVs and LDCVs in DRG neurons. We found that while CAPS1 promotes SV exocytosis, CAPS2 is responsible for LDCV fusion (summarized in Figure 8). The distinct functions of the CAPS paralogs are not due to specificity in their molecular interactions with distinct vesicular proteins located on either LDCVs or SVs. In fact, exogenous expression of each CAPS paralog similarly rescued LDCV exocytosis in CAPS DKO neurons. Instead, their specificity is likely based on their sub-cellular localization. In WT DRG neurons, CAPS1 concentration was highly increased at synapses compared to adjacent neurites and soma. This finding compares well with distribution of CAPS1 in hippocampal neurons (Farina et al., 2015). In contrast to the highly punctate staining of CAPS1, CAPS2 localization in DRG neurons is diffuse and primarily somatic. As their differential function is due to their specific localization, the subcellular transport mechanism of both CAPS paralogs must differ and be tightly controlled. In the peripheral nervous system, the cell soma and synaptic contacts are separated by a great distance, which poses a challenge for synaptic targeting of cytoplasmic proteins. Another priming factor, Munc13, overcomes this problem by its association with so-called piccolo/bassoon transport vesicles (Ohtsuka et al., 2002; Maas et al., 2012). It is possible that CAPS1 utilizes the same means of transportation while CAPS2 does not.

The number of LDCVs released at the soma visualized by TIRFM was relatively low in comparison to what can be measured in the neurites of hippocampal neurons. However, release of NPY-Venus from LDCVs was clearly identifiable due to its pH-sensitivity. Hence, we are confident that we do not miss a significant number of fusion events. This is supported by the fact that after converting LDCV fusion in fusion probability, we found that it was twice as high as in hippocampal neurons. It also shows that we can reliably discern between secreting and not secreting cells. Additionally, we measured a large number of cells and we partially blindly evaluated the data. Thus, although LDCV exocytosis appears close to detection level, we are convinced that we reliably demonstrated that at the DRG neuron soma LDCV exocytosis is only promoted by CAPS2. Somatic liberation of neuropeptides has been reported to be essential for pain and neuropathic pain sensation. In these pathological conditions, “spike activity in excited DRG neurons induces subthreshold depolarization and excitation in their passive neighbors” (Devor, 1999). This well-documented phenomenon, termed cross-excitation, has been attributed to somatic liberation of NPY and other neuropeptides (Spigelman and Puil, 1990; Brumovsky et al., 2007; Omoto et al., 2015). Therefore, CAPS2-regulated exocytosis of LDCV at the soma is highly relevant.

LDCVs are also secreted in neurites and in synapses (Xia et al., 2009; van de Bospoort et al., 2012). Our data did not unambiguously identify which CAPS paralog mediates neuropeptide release close to the synapses in DRG neurons. In excitatory hippocampal neurons, LDCV fusion near synaptic sites is mediated by CAPS1 (Farina et al., 2015), but the expression level of CAPS2 is very low in these cells (Jockusch et al., 2007). In inhibitory hippocampal neurons, where the expression pattern of CAPS1 and CAPS2 is inverted (CAPS1 absent and high concentration of CAPS2), LDCV fusion in neurites is mediated by CAPS2 (Shinoda et al., 2011). This indicates that it is the availability of CAPS paralogs that dictates their function. Because CAPS1 is highly enriched at synapses in comparison to CAPS2, our data might imply that CAPS1 promotes LDCV exocytosis near synapses in DRG neurons. However, we found that inhibition of secreted neuropeptides and deletion of CAPS2, but not deletion of CAPS1 led to more synchronized SV fusion in DRG neurons. Accordingly, CAPS2-dependent release of neuropeptides indirectly regulates SV exocytosis. If CAPS1 drives peri-synaptic neuropeptide secretion and CAPS2 promotes somatic release, deletion of CAPS2 may alter the global extracellular concentration of neuropeptides, but their concentration near the synaptic cleft should remain largely unaltered. Thus, CAPS2 deficiency should not affect neuropeptide-dependent synchronization of synaptic activity to the same extent as CAPS DKO. As our data indicated the inverse, we suggest that CAPS2 likely mediates neuropeptide release near synapses and induces non-synchronized synaptic transmission (Figure 8). This scenario requires precise localization of CAPS1 at the active zone rather than the entire presynaptic site.

We showed that iB_4_-positive cells are largely unable to release neuropeptides and that these cells do not express CAPS2. Because overexpression of CAPS2 in these cells promoted neuropeptide release, one might speculate that CAPS2 is the only missing factor in non-peptidergic neurons to release neuropeptides. However, recent studies using single cell RNA-sequencing showed that iB_4_ staining is not as reliable as desirable to classify DRG neurons in peptidergic and non-peptidergic neurons (Usoskin et al., 2015; Li et al., 2016). Hence, another hypothesis would be that some iB_4_-positive neurons are in fact peptidergic neurons that use another priming factor such as Munc13 to release neuropeptides. That scenario, however, does not explain why these cells do not release neuropeptides upon our stimulation protocol. Because the protein expression pattern of DRG neurons is strongly dependent on their physiological state (Xiao et al., 2002; Bangash et al., 2018), another possibility is that some peptidergic neurons are transiently not expressing CAPS2 and are thus incapable to secrete neuropeptides. But would this hypothesis fit with our data? A comprehensive study, in which single cell RNA-sequencing was combined with functional assays and iB_4_ staining, showed that about 20% of the iB_4_-positive DRG neurons expressed CGRP and that some additional overlap between iB_4_ staining and SP expression existed (Li et al., 2016). Similarly, we found 11% overlap between SP and iB_4_ labeling (Supplementary Figure S1A). However, none of the iB_4_-positive neurons expressed any other neuropeptide such as NPY, neuropeptide B, neuromedin B, galanin, somatostatin etc. (Li et al., 2016). Overall, about 30% iB_4_-positive neurons were incorrectly identified as non-peptidergic in that study. Since we found that overexpression of CAPS2 in WT iB_4_-positive cells increased the percentage of secreting cells from 3% to 60%, an incorrect identification of peptidergic neuron could only partially explain our results. Therefore, it seems more likely that CAPS2 overexpression in WT iB_4_-positive cells promotes exocytosis of LDCVs in non-peptidergic neurons.

Overexpressing any paralog of CAPS in CAPS DKO neurons using Semliki Forest virus not only rescued LDCV exocytosis, but also amplified the response compared to that in WT DRG neurons. In chromaffin cells, LDCV exocytosis is not substantially increased upon CAPS1 or CAPS2 overexpression (Liu et al., 2010). Similarly, synaptic transmission and LDCV exocytosis in CAPS DKO hippocampal neurons was rescued only to the level of such observed in WT neurons upon Semliki Forest virus-driven CAPS1 overexpression (Jockusch et al., 2007; Farina et al., 2015). Finally, in cerebellar granule cells, overexpression of CAPS2 increased BDNF or neurotrophin 3 secretion by a factor of two (Sadakata et al., 2004). Therefore, unlike that in chromaffin cells or hippocampal neurons, but similar to that in cerebellar granule cells, the CAPS2 expression level in DRG neurons is not saturated, allowing for precise regulation of neuropeptide release. A variety of studies have demonstrated that neuropeptide secretion is enhanced when neuropathic pain is established (Garry and Hargreaves, 1992; Collin et al., 1993). One possible mechanism is that induction of neuropathic pain is accompanied by enhanced CAPS2 synthesis in peptidergic DRG neurons, thereby increasing neuropeptide secretion. Alternatively, neuropathic pain could also increase the overall number of neurons secreting neuropeptides. Axotomy or nerve ligation induces *de novo* expression of numerous neuropeptides in DRG neurons devoid of these peptides prior to surgery (Nitzan-Luques et al., 2013). For some neuropeptides such as NPY and galanin, their expression is not only induced in peptidergic neurons but also in non-peptidergic neurons (Boateng et al., 2015). We demonstrated that non-peptidergic neurons are devoid of CAPS2 and are unable to release neuropeptides. Hence, nerve lesion should not only induce peptide synthesis but also trigger CAPS2 expression in these neurons. Interestingly, this increased expression of neuropeptides and CAPS2 might be interdependent, because CAPS2 appears to be involved in LDCV biogenesis and trafficking via its interaction with class II ADP ribosylation factor small GTPases (Sadakata et al., 2012). Consequently, CAPS2 might play a pivotal role by upregulating LDCV biogenesis and exocytosis after nerve lesion.

## Conclusion

This study demonstrates for the first time that different paralogs of the same cytoplasmic priming factor specifically regulate exocytosis of two different vesicle types due to their subcellular localization. This may be a unique feature of DRG neurons, as it might be related to their shape and axon length or to the fact that both types of vesicle play an important role in signal transduction. In the first scenario, CAPS paralogs should behave as they do in motor neurons, in which the soma and synaptic contacts are also separated by a great distance. In the second scenario, neurons such as striatal neurons, whose neurotransmitter and neuropeptide release are of equal importance, should utilize similar mechanisms to differentially regulate their secretion. Finally, it will be of great interest to study whether this behavior is restricted to CAPS paralogs or if paralogs of other priming factors such as Munc13 follow the same paradigm. Considering the importance of peptide release in neuropathic pain, our findings open novel avenues for research on the molecular mechanism of neuropathic pain.

## Author Contributions

UB and AS: conceptualization. AS, AH, MK, RM, CS and UB: methodology. AS, AStaudt, AShaaban, AH, CS and UB: investigation. UB, AS, RM and JR: writing. UB and JR: supervision.

## Conflict of Interest Statement

The authors declare that the research was conducted in the absence of any commercial or financial relationships that could be construed as a potential conflict of interest.

## Footnotes

^1^http://imagej.nih.gov/ij/
