# Supplementary material for: Paralogs of the Calcium-Dependent Activator Protein for Secretion Differentially Regulate Synaptic Transmission and Peptide Secretion in Sensory Neurons
Source: Front Cell Neurosci. 2018 Sep 11;12:304. doi: 10.3389/fncel.2018.00304 (PMC6141663; doi:10.3389/fncel.2018.00304)
Supplement: Supplementary file 1 [file Data_Sheet_1.pdf]

Paralogs of the calcium-dependent activator protein for secretion differentially regulate synaptic transmission and peptide secretion in sensory neurons

Ali H. Shaib, Angelina Staudt, Ali Harb, Margarete Klose, Ahmed Shaaban, Claudia Schirra, Ralf Mohrmann, Jens Rettig and Ute Becherer

Supplementary figures 1 – 6.

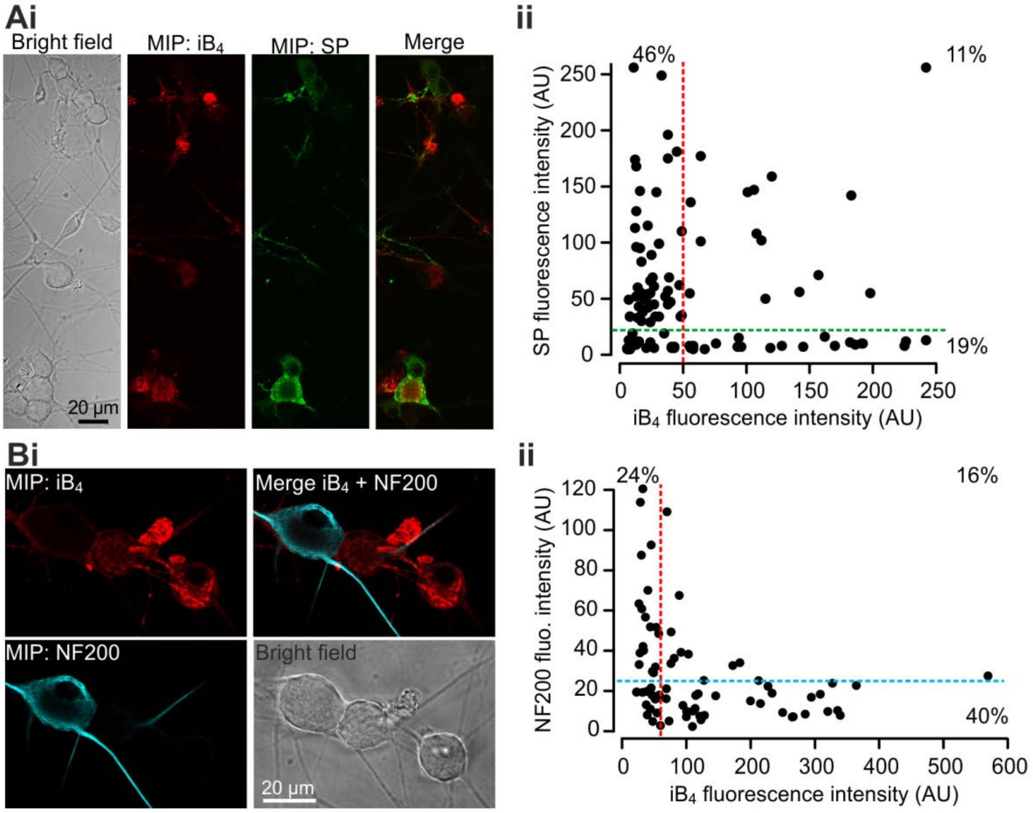

Figure S1: iB4 staining allows the identification of peptidergic neurons

(A) iB_4 and anti-substance P (SP/TAC1) labelling **(Ai)** Maximum intensity projection (MIP) of confocal images of DRG neurons that were labeled with anti-TAC1 antibody (green) and iB_4-Alexa561 (red). One neuron is co-stained with the anti-TAC1 antibody and iB_4 and one neuron is not stained at all. But a majority are stained by either iB_4 or anti-TAC1 antibody. **(Aii)** Correlation plot between the fluorescence intensity of iB_4 and anti-TAC1 antibody labeling. Threshold to distinguish between cells that are positive or negative for iB_4 or anti-TAC1 staining were determined with a distribution histogram and are represented as red and green stippled lines. Few cells cannot be unequivocally classified because they were clearly iB_4 and NF200 positive cells. Additionally 24% of the cells were iB_4 and anti-TAC1 negative. These neurons might express other peptides such as CGRP and are likely peptidergic ($n_{neurons}$=107). **(B)** iB_4 and anti-NF200 labelling. **(Bi)** MIP of DRG neurons double labeled with anti-NF200 antibody (cyan) and iB_4-Alexa561 (red). No co-staining is visible indicating that peptidergic neurons (iB_4 negative and NF200 positive) can be distinguished from non-peptidergic neurons (iB_4 positive and NF200 negative) using iB_4 staining. **(Bii)** Correlation plot between the fluorescence intensity of iB_4 labeling and NF200 antibody staining. A distribution histogram was generated to determine the threshold fluorescence between cells that are positive or negative for iB_4 or NF200. The threshold are represented as red and blue stippled lines. Few cells cannot be unambiguously classified as they were clearly iB_4 and NF200 positive, and about 18% of the cells were iB_4 and NF200 negative ($n_{neurons}$=75).

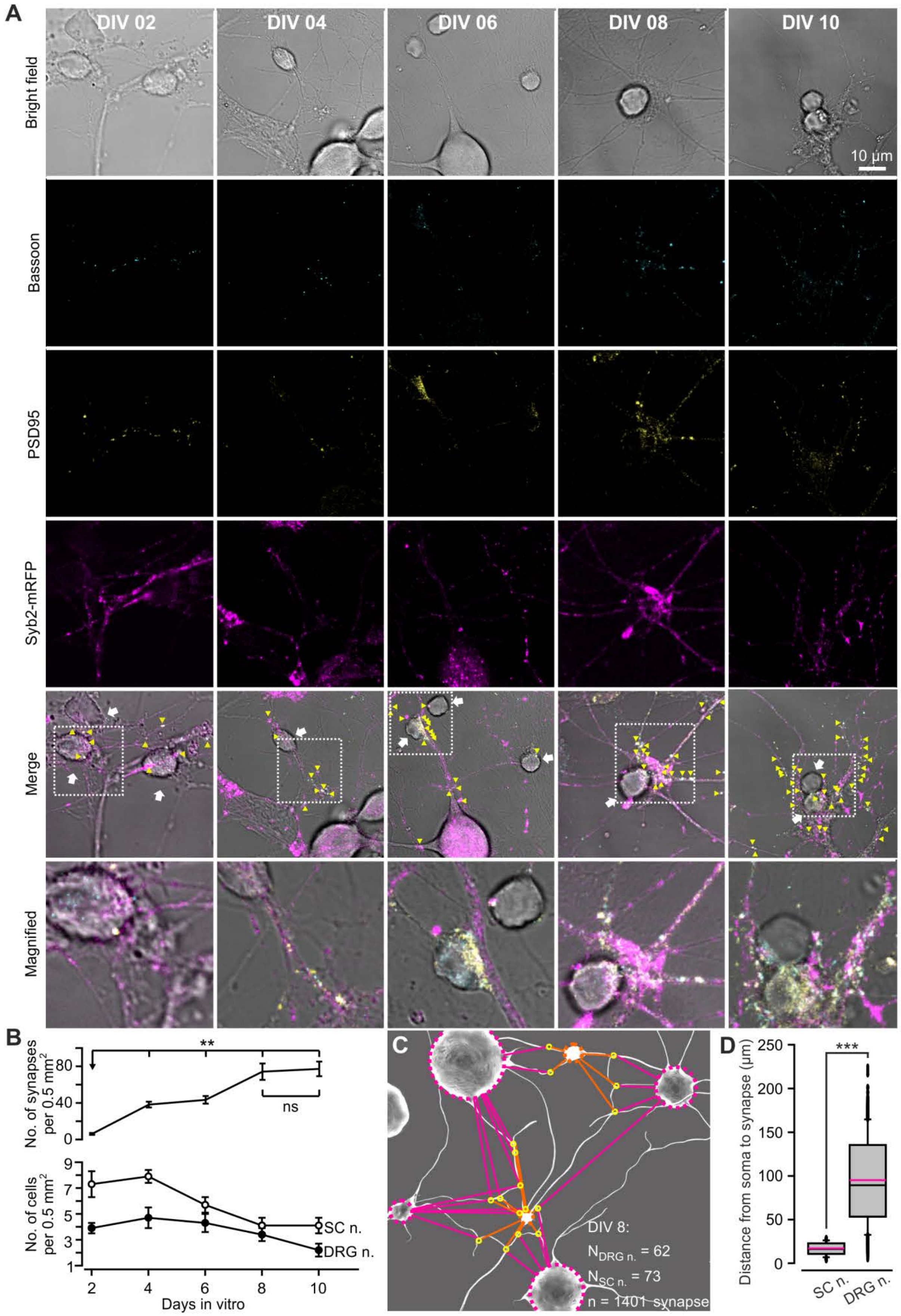

Figure S2. Timeline of synapse formation between DRG and SC neurons.

Co-cultured Syb2-mRFP Knock-In DRG neurons with WT SC neurons were fixed with 4% PFA at DIV 2, 4, 6, 8 and 10 after addition of SC neurons. Syb2-mRFP in DRG neurons allowed the identification of heterotypic synapses that were formed between DRG and SC neurons and the exclusion of SC homotypic synapses. To identify functional synapses, neurons were stained against presynaptic marker bassoon and postsynaptic marker PSD-95. **(A)** From top to bottom bright field and confocal images of bassoon (cyan), PSD-95 (yellow) and Syb2-mRFP (magenta) labelling. The fifth row of pictures corresponds to the overlay of all 4 channels. White arrows point at SC neurons and yellow arrows indicate synapses in which bassoon, PSD-95 and Syb2-mRFP signals co-localize. Images in the last row are magnified regions (stippled line) of the pictures shown in row 5. **(B)** Top: Average number of synapses over time in which bassoon, PSD-95 and the Syb2-mRFP signal co-localized. Bottom: number of living DRG and SC neurons over time. The data were normalized to the acquired surface area. **(C)** Schematic representation of the analysis method used to measure the distance from each heterotypic synapse to its DRG neuron and the nearest SC neuron cell body. **(D)** Box plot of the distance between heterotypic synapses and neuronal cell bodies at DIV 8. Pink and black lines in the box correspond to the average and median distances, respectively. DRG neurons were isolated from 2 adult mice while SC neurons were isolated from 12 P0 mice making 2 separate cultures. DIV 2, $n_{DRG\ neurons} = 75$, $n_{SC\ neurons} = 139$ and $n_{synapses} = 286$; DIV 4, $n_{DRG\ neurons} = 84$, $n_{SC\ neurons} = 157$ and $n_{synapses} = 258$; DIV 6, $n_{DRG\ neurons} = 82$, $n_{SC\ neurons} = 109$ and $n_{synapses} = 845$; DIV 8, $n_{DRG\ neurons} = 62$, $n_{SC\ neurons} = 73$ and $n_{synapses} = 1401$ and DIV 10, $n_{DRG\ neurons} = 31$, $n_{SC\ neurons} = 58$ and $n_{synapses} = 1120$. Error bars are SEM , ***$p < 0.001$, ** $p < 0.01$, and ns (not significant) $p > 0.05$, one-way ANOVA Dunn's test (B) and Students t-test (D).

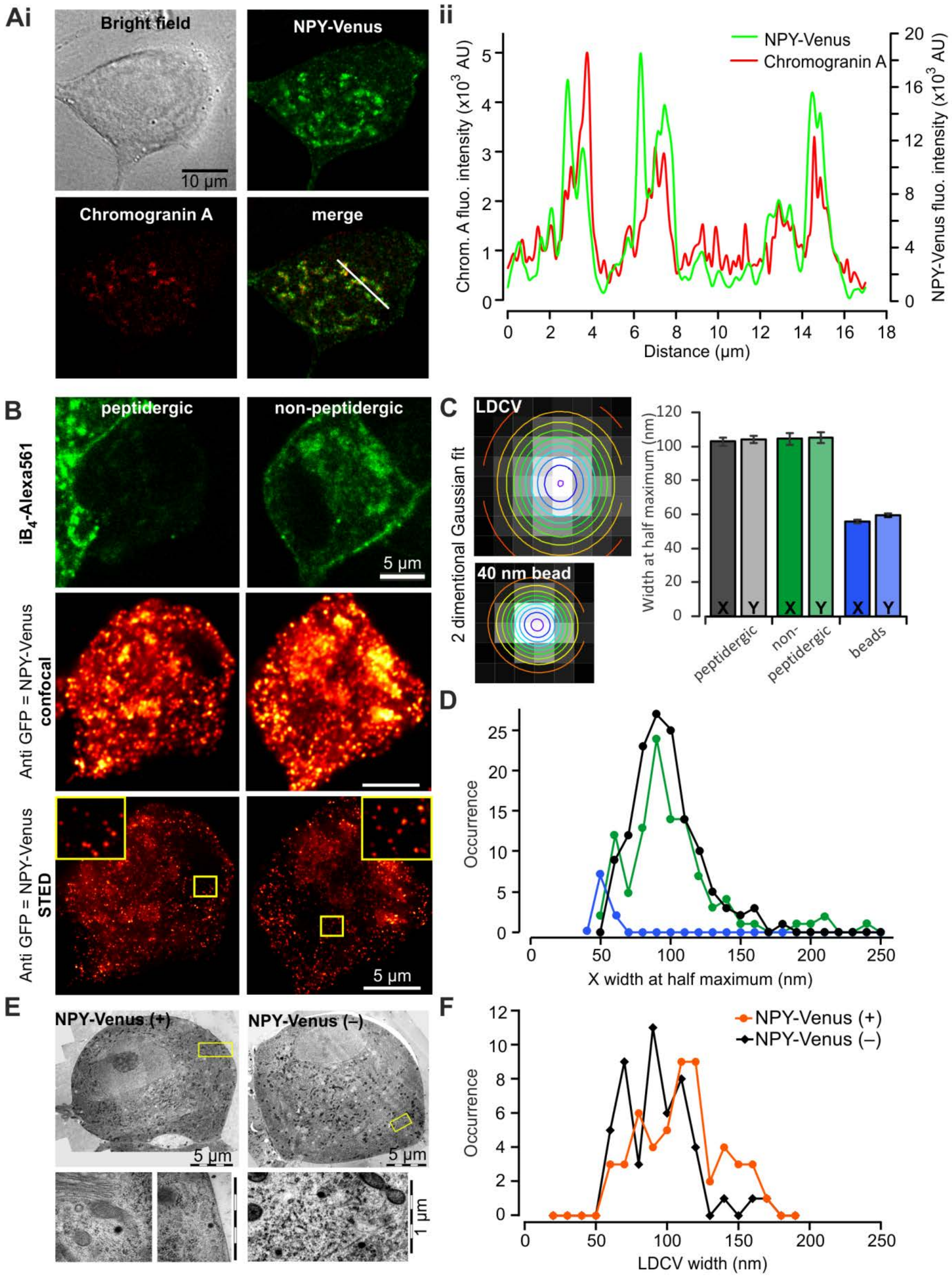

Figure S3: NPY-Venus is localized to LDCVs in peptidergic and non-peptidergic neurons.

(A) NPY-Venus co-localizes with chromogranin A in peptidergic neurons. Representative picture of a DIV 7 old DRG neuron transfected with NPY-Venus, fixed with 4% PFA, and subsequently permeabilized with 0.2% triton. The cells were incubated overnight with the anti-chromogranin A and stained with a goat anti-rabbit Alexa561 antibody. **(Ai)** Bright field and confocal images of a DRG neuron acquired at 514 nm for NPY-Venus and 561 nm for Chromogranin A. **(Aii)** Line scan (see overlay image in Ai) analysis reveals co-localization of NPY with Chromogranin A at the level of individual vesicles. Similar results were obtained in 46 cells from 2 adult mice.

(B) LDCVs marked with NPY-Venus in peptidergic and non-peptidergic neurons measured with STED microscopy. Top: MIPs of confocal images from peptidergic (left) and non-peptidergic (right) neurons identified through iB_4–Alexa647 staining. Middle: single plane confocal images of NPY-Venus labeling in these cells. Because Venus bleaches to fast to be imaged in STED microscopy, it was counterstained with immunocytochemistry using anti-GFP antibody and anti-rabbit STAR red secondary antibody. Bottom: matching single plane STED images of these cells. Insets correspond to enlarged portions of the images delineated in yellow. **(C)** Graph of the X and Y full width at half maximum of vesicles from peptidergic and non-peptidergic neurons. Vesicles labeled with NPY-Venus had a diameter of 103 ± 2 nm in both DRG neuron subtypes ($n_{neurons}$ = 7 and 6, n_{LDCVs} = 135 and 105 for peptidergic and non-peptidergic, respectively). To insure that we had proper resolution to distinguish variations in vesicle size, we imaged 40 nm large crimson red beads (Invitrogen, bottom picture) with the same STED settings as used to image DRG neurons. Their apparent size was 55.6 ± 1.1 nm well below the size of NPY-Venus labelled vesicles (n=10). **(D)** The size distribution of NPY-Venus labeled vesicles show that they represent one population whether in peptidergic or non-peptidergic neurons. **(E)** Electron microgram of DRG neurons transfected (left) or not (right) with NPY-Venus. The expression of NPY-Venus was verified in a CLEM experiment as described in (Matti et al., 2013). Top row shows an overview of the entire neuron while the bottom row shows enlarged exemplary LDCVs. Their diameter was measured by surrounding LDCVs with elliptical regions of interest and using the ImageJ function "fit ellipse". **(F)** Size distribution of LDCVs in DRG neurons expressing (orange) or not (black) NPY-Venus. Overexpression of NPY-Venus increased the LDCV diameter from 97.7 ± 3.5 nm in WT control cells to 115.7 ± 3.9 nm in transfected cells (n_{LDCVs} = 49 and 51, respectively, p=0.002 (Student t-test)). LDCV diameter measured on the electron micrograms corresponds very well to the size measured on images acquired with STED microscopy shown in (C).

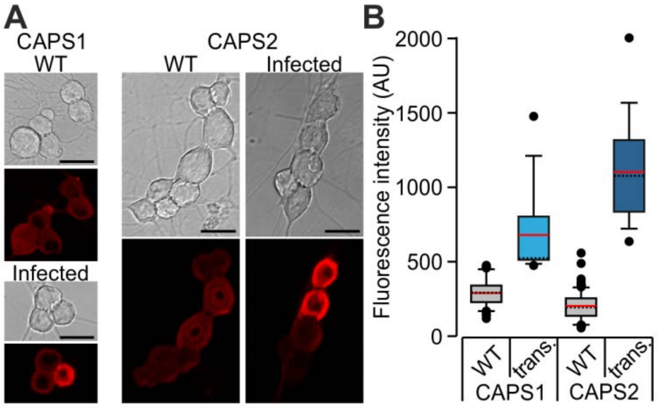

Figure S4: CAPS 1 or CAPS2 overexpression via Semliki Forest virus lead to at least 3 fold increased expression of the protein in comparison to its level in WT control cells.

(A) WT DRG neurons were transfected via Semliki Forest virus encoding either for CAPS1 or CAPS2. The cells were incubated with the virus for 5.5 h before fixation. The transfection efficacy was approximately 15% and 30% for the virus encoding for CAPS1 and CAPS2, respectively. The cells were fixed and stained with anti-CAPS1 (left) or anti-CAPS2 antibody (right). They were imaged using confocal microscopy. Shown are cells that were not incubated with the virus (WT) and cells that were incubated with the virus (infected). Scale bars are 20 µm. **(B)** Box plot displaying the fluorescence intensity of the antibody staining to evaluate the level of CAPS expression in transfected (trans, blue bars) and control cells (WT, gray bars). The stippled line is the median while the red line represents the mean.

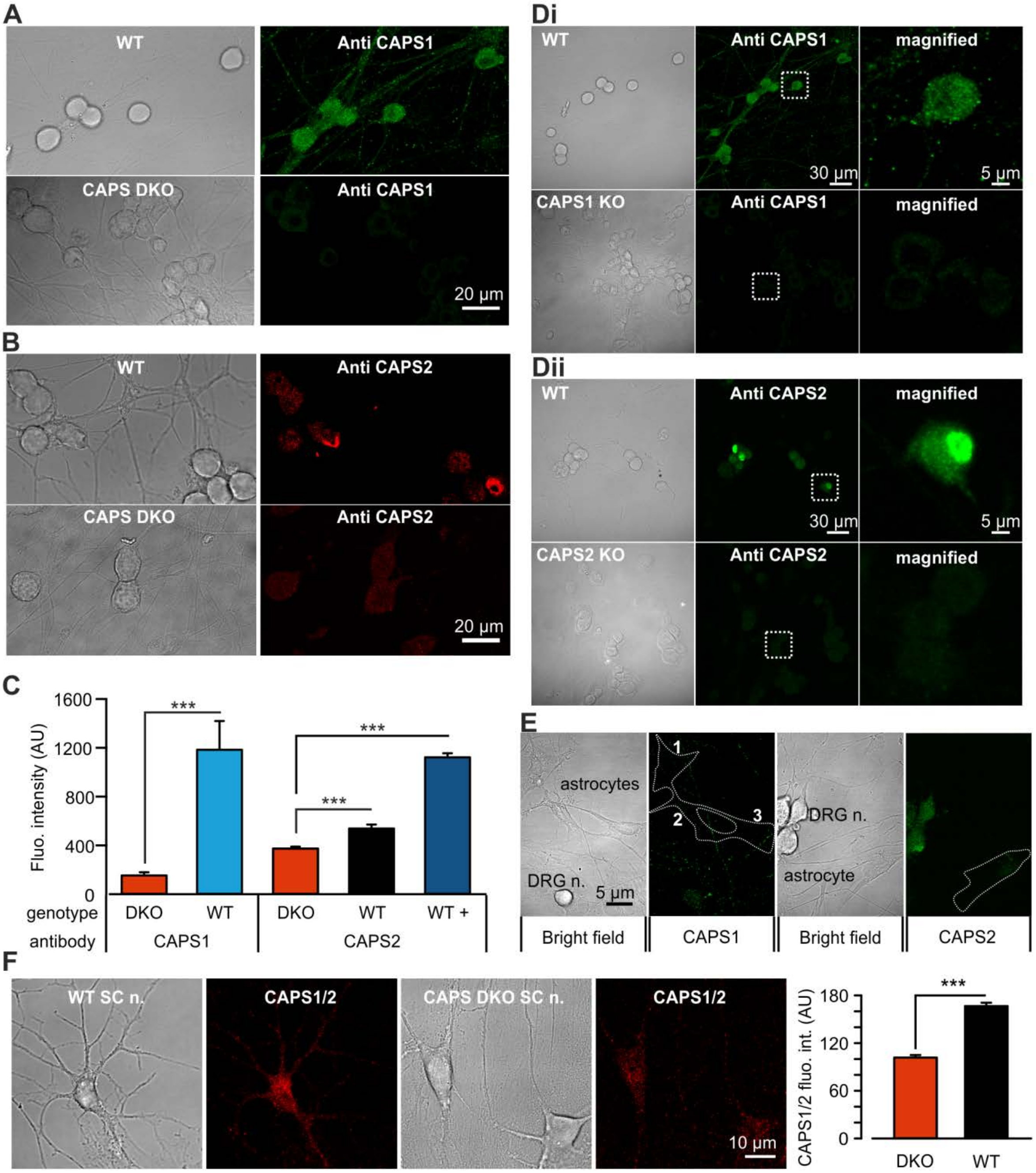

Figure S5: anti-CAPS1 and anti-CAPS2 antibodies are specific.

(A) Bright field (right) and confocal (left) images of WT (left) and CAPS DKO (right) DRG neurons stained with anti-CAPS1 antibody. **(B)** Bright field (right) and confocal (left) images for WT (left) and CAPS DKO (right) DRG neurons stained with anti-CAPS2 antibody. **(C)** Quantification of fluorescence intensity of anti-CAPS1 or anti-CAPS2 staining in WT DRG neurons compared to CAPS DKO DRG neurons. Because CAPS2 is expressed almost exclusively in peptidergic DRG neurons (Figure S3), CAPS2 fluorescence intensity was displayed at a global average of all WT neurons (WT) and as average of CAPS2 positive WT neurons (WT+). The same cultures were used for both staining hence N_{mice} equals 3 for both adult WT mice and E18 CAPS DKO embryos. Analysis of anti-CAPS1 staining was performed on 32 and 90 WT and DKO neurons, respectively. For anti-CAPS2 labeling 28 and 94 WT and DKO neurons were measured, respectively. Mann Whitney significance test was applied, *** $p < 0.001$. **(D)** To ensure no cross-reactivity of the antibodies against the other paralog, anti-CAPS1 antibody specificity was tested in WT and compared to CAPS1 KO neurons (**Di**) and anti-CAPS2 antibody was tested in WT and compared to CAPS2 KO neurons (**Dii**). **(E)** Astrocytes exhibited little CAPS1 or CAPS2 staining. **(F)** SC WT neurons were stained against CAPS1 and CAPS2 using in house serum that detects both paralogs. N_{mice} = 2 E18 embryos, n_{WT} = 83 and n_{DKO} = 62 neuron. Error bars are SEM and ***$p < 0.001$, Mann Whitney test.

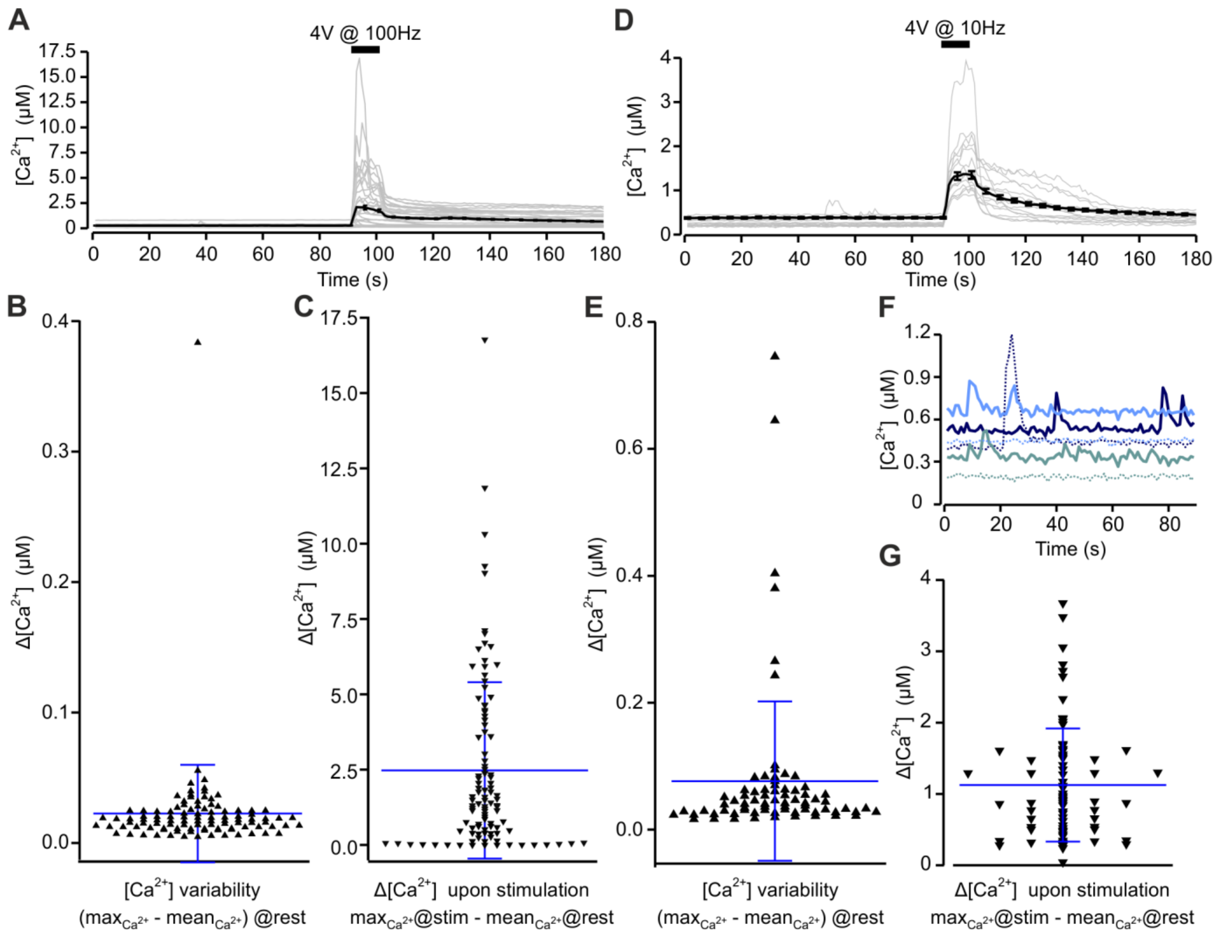

Figure S6: DRG neurons in culture do not show network activity.

(A - C) $[Ca^{2+}]_i$ was recorded from a total of 103, 7 DIV old DRG neurons. The cells were maintained 90 s at rest to measure spontaneous $[Ca^{2+}]_i$ oscillation then they were stimulated for 10 s at 4 V and 100 Hz with the field electrode to assess the stimulus efficacy applied to evoke LDCV exocytosis. **(A)** Time course of $[Ca^{2+}]_i$. Gray traces are exemplary recordings of 26 individual cells while the black trace is the average of all recorded cells (n=103 ± SEM). The cells are essentially silent before stimulus and 4 V stimulus elicited a reliable $[Ca^{2+}]_i$ increase in a majority of DRG neurons. **(B)** Dot plot representing the maximum $[Ca^{2+}]_i$ fluctuation before stimulus calculated as maximum resting $[Ca^{2+}]_i$ minus average resting $[Ca^{2+}]_i$. Only one cell out of 103 showed a marked $[Ca^{2+}]_i$ increase. All other cells had $[Ca^{2+}]_i$ variations of less than 100 nM. **(C)** Dot plot of the maximum $[Ca^{2+}]_i$ increase upon electrical stimulation reveals that more than 80 % of the cells responded to the stimulus with a significant $[Ca^{2+}]_i$ increase. The threshold was 222 nM above resting level that corresponds to 4 times the average $[Ca^{2+}]_i$ fluctuation at rest shown in B. **(D - G)** $[Ca^{2+}]_i$ was recorded from a total of 81 DRG neurons maintained 8 DIV in co-culture with SC neurons. Spontaneous $[Ca^{2+}]_i$ oscillations were measured for 90 s in resting condition, then the DRG neurons were stimulated for 10 s at 4 V and 10 Hz with the field electrode to assess the stimulus efficacy applied to evoke synaptic transmission. **(D)** Time course of $[Ca^{2+}]_i$. Gray traces are representative recordings of 14 individual cells while the black trace is the average of all recorded cells (n = 81 ± SEM). **(E)** Dot plot representing $[Ca^{2+}]_i$ fluctuations at rest in all measured DRG neurons. Resting $[Ca^{2+}]_i$ is clearly less stable in DRG neurons that were maintained in co-culture as compared to those maintained alone as seen in panel B (note the scale difference between B and E). However, less than 15% of the neurons displayed $[Ca^{2+}]_i$ fluctuations above 100 nM. **(F)** These $[Ca^{2+}]_i$ oscillations were not due to network activity since they could not be inhibited by the co-application of two glutamate antagonists D-2-amino-5-phosphonopentanoate (50 µM, APV) and DNQX (25 µM). Shown are the $[Ca^{2+}]_i$ recordings at rest of three cells that displayed spontaneous activity prior (stippled line) and/or after (solid line) antagonists application. **(G)** Dot plot of the maximum $[Ca^{2+}]_i$ increase upon electrical stimulation reveals that more than 77% of the cells responded to the stimulus with a significant $[Ca^{2+}]_i$ increase. The threshold was the same as in C. **(B, C, E & G)** Blue lines are the average ± SD.
